# Mitochondrial dysfunction–induced H3K27 hyperacetylation perturbs enhancers in Parkinson’s disease

**DOI:** 10.1172/jci.insight.138088

**Published:** 2021-09-08

**Authors:** Minhong Huang, Dan Lou, Adhithiya Charli, Dehui Kong, Huajun Jin, Gary Zenitsky, Vellareddy Anantharam, Arthi Kanthasamy, Zhibin Wang, Anumantha G. Kanthasamy

**Affiliations:** 1 Parkinson Disorders Research Laboratory, Iowa Center for Advanced Neurotoxicology, Department of Biomedical Sciences, Iowa State University, Ames, Iowa, USA.; 2 Laboratory of Environmental Epigenomes, Department of Environmental Health and Engineering, Bloomberg School of Public Health, Johns Hopkins University, Baltimore, Maryland, USA.; 3 State Key Laboratory of Biocatalysis and Enzyme Engineering, College of Life Sciences, Hubei University, Wuhan, Hubei Province, China.

**Keywords:** Neuroscience, Epigenetics, Mitochondria, Parkinson disease

## Abstract

Mitochondrial dysfunction is a major pathophysiological contributor to the progression of Parkinson’s disease (PD); however, whether it contributes to epigenetic dysregulation remains unknown. Here, we show that both chemically and genetically driven mitochondrial dysfunctions share a common mechanism of epigenetic dysregulation. Under both scenarios, lysine 27 acetylation of likely variant H3.3 (H3.3K27ac) increased in dopaminergic neuronal models of PD, thereby opening that region to active enhancer activity via H3K27ac. These vulnerable epigenomic loci represent potential transcription factor motifs for PD pathogenesis. We further confirmed that mitochondrial dysfunction induces H3K27ac in ex vivo and in vivo (MitoPark) neurodegenerative models of PD. Notably, the significantly increased H3K27ac in postmortem PD brains highlights the clinical relevance to the human PD population. Our results reveal an exciting mitochondrial dysfunction-metabolism-H3K27ac-transcriptome axis for PD pathogenesis. Collectively, the mechanistic insights link mitochondrial dysfunction to epigenetic dysregulation in dopaminergic degeneration and offer potential new epigenetic intervention strategies for PD.

## Graphical abstract

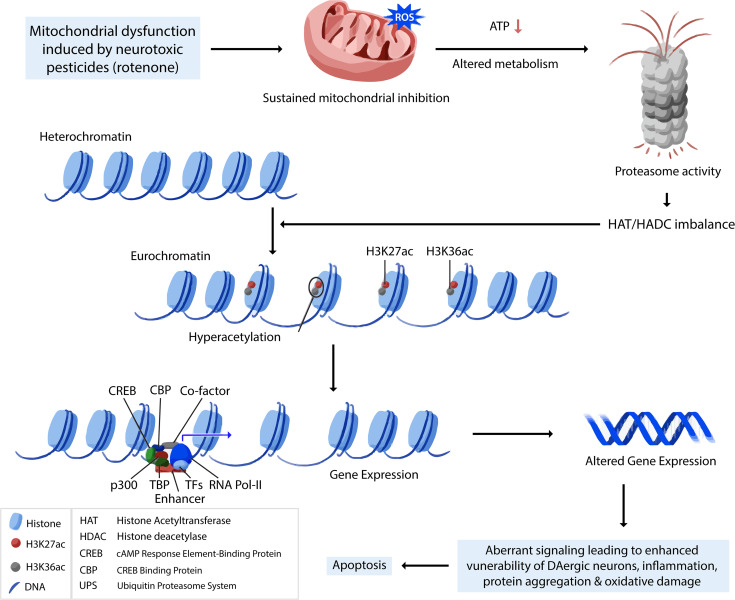

## Introduction

Since the first reports of *SNCA* mutation in the pathogenesis of Parkinson’s disease (PD) (1), investigations on genetic causes in PD have flourished and gained mainstream attention in the scientific community. However, genetic mutations at 15 loci explain only about 10%–15% of PD cases (2, 3). Notably, mutations of several key PD genes, including *PARK2* (Parkin), *PARK6* (PINK1), *SNCA*, and *LRRK2*, modify key mitochondrial functions (4, 5).However, environmental exposure and its interactions with genetic factors are expected to explain most PD cases. Many environmental neurotoxicants are known to impair mitochondrial functions. For example, the pesticide rotenone inhibits mitochondrial functionality by impairing oxidative phosphorylation and protein synthesis, leading to energy depletion (6, 7). Thus, mitochondrial dysfunction is key for both genetically and environmentally linked PD; however, the underlying mechanism remains unclear.

Recent studies imply that epigenomic alteration plays an important role in the neuropathology and etiology of PD (8). We previously discovered that neurotoxic pesticides induce the acetylation of core histones and apoptotic cell death in dopaminergic (DAergic) cell culture (9, 10). Increased acetylation of core histones in an animal model of PD and in human PD brains has been reported (11). Hyperacetylation makes neurons vulnerable to environmental toxicity, thereby increasing the risk of developing PD (9, 10, 12). However, the detailed mechanism of neurotoxic pesticide–induced histone modification and its core role in environmentally linked PD remain unclear.

Among many histone acetylation (HAc) marks, H3K27ac is a robust mark of active promoters and distal enhancers that are tightly coupled to gene expression and transcription factor (TF) binding (13–15). By marking the genome, H3K27ac acts as an epigenomic memory of environmental stimuli and mediates a stronger response upon subsequent stimulation. Aberrant H3K27ac modification triggered by environmental stimuli would consequently induce pervasive changes in gene expression. Altered gene expression is considered to be a fundamental hallmark of environmentally linked PD (16–18). Comprehensive genome-wide analysis of gene expression alterations and H3K27ac modifications can better elucidate the complex cooperative biological processes occurring in the genome during PD pathogenesis. However, such high-resolution analysis has never been performed in PD models to our knowledge, and the pattern by which H3K27ac modification responds to environmental stimuli and mitochondrial impairment to affect PD pathogenesis remains to be elucidated.

Here, we characterized H3K27 acetylation modification and mRNA transcription levels at certain genomic loci in multiple PD models, including DAergic neuronal cell cultures, ex vivo nigral brain slice culture, in vivo mouse models (MitoPark [MP]), and postmortem human PD brains. In addition, by integrated bioinformatics analysis of ChIP-Seq and RNA-Seq on mitochondria-impaired DAergic neuronal models, we identified the vulnerable epigenomic loci regulated by H3K27 hyperacetylation in both environment-linked and mitochondrial genetic mutation–linked DAergic neurodegeneration.

## Results

### Genome-wide hyperacetylation profiling identifies H3K27ac as a key lysine acetylation site in mitochondria-impaired DAergic neuronal cells.

Our previous studies (9, 10) demonstrated for the first time to our knowledge that exposure to neurotoxic pesticides induces global acetylation of core histones H3/H4, and that pharmacological inhibition of histone hyperacetylation protects against neurotoxic pesticide–induced DAergic neurotoxicity. However, the exact patterns of HAc and its mechanism upon mitochondrial dysfunction remain to be elucidated (19, 20). To this end, we exposed DAergic N27 neuronal cells to the mitochondrial complex I inhibitor rotenone in a low-dose exposure paradigm (1 μM for 3 hours) and performed a quantitative histone acetylome analysis to map the lysine acetylation (Kac) sites of the core histones.

Although short-term rotenone exposure was sufficient to impair mitochondrial function, it only induced a small time-dependent decline in viability not exceeding 10% (Supplemental Figure 1; supplemental material available online with this article; https://doi.org/10.1172/jci.insight.138088DS1). We then characterized and confirmed its mitochondrial dysfunction. Our JC-1 assay shows a decreased red/green ratio in the rotenone-treated cells (Supplemental Figure 2, A and B), indicative of increased depolarization of the mitochondrial membrane. MitoTracker probe staining revealed increased mitochondrial circularity and damaged mitochondrial structure in rotenone-treated cells (Supplemental Figure 2, C and D). The functional consequences of the mitochondrial damage emerged as reductions in basal respiration rate, spare respiratory capacity, proton leak, and ATP production in the rotenone-treated cells, as revealed by extracellular flux analyses (Supplemental Figure 2, E–G). A cell energy phenotype test shows that rotenone also stressed metabolic activity as measured by the oxygen consumption rate and extracellular acidification rate/glycolysis (Supplemental Figure 2H). Moreover, oxidative stress was induced in rotenone-treated N27 cells as indicated by markedly increased mitochondrial ROS production in the MitoSox assay (Supplemental Figure 2, I and J). Next, from our AcetylScan method, among 3165 (out of 3171) Kac peptides, the fold changes of which exceeded 2.5-fold, we found that histone H3 exhibited substantially higher fold changes in mitochondria-impaired N27 neuronal cells than in untreated cells. Consistent with our previous data (9, 10) as well as published studies (21, 22), these results suggest that PD-related mitochondrial impairment induces H3 hyperacetylation. A histone H3 peptide containing di-acetylated lysine sites at both K27 and K36 displayed the highest HAc increase (99.2-fold) compared with control (Table 1, nos. 1 and 2). To eliminate the possibility that the highest peak of this peptide was attributable to the K36ac site rather than K27ac, we further tested the peptide of a single H3K36ac site (Table 1, no. 3). H3K36ac increased only 4.9-fold, whereas another histone H3 peptide containing both K27 and K36 ac sites from histone variant H3.3B increased 47.1-fold, indicating that acetylation of K27, but not that of K36, causes the highest increase in HAc detected and may contribute to the corresponding gene expression reprofiling. Since H3K27ac and H3K36ac peaked together in both peptide sequences, H3K36ac likely facilitates H3K27ac to produce distinct outcomes in response to neurotoxicity, rather than H3K27ac alone reacting to cellular stress.

**Figure 1:**
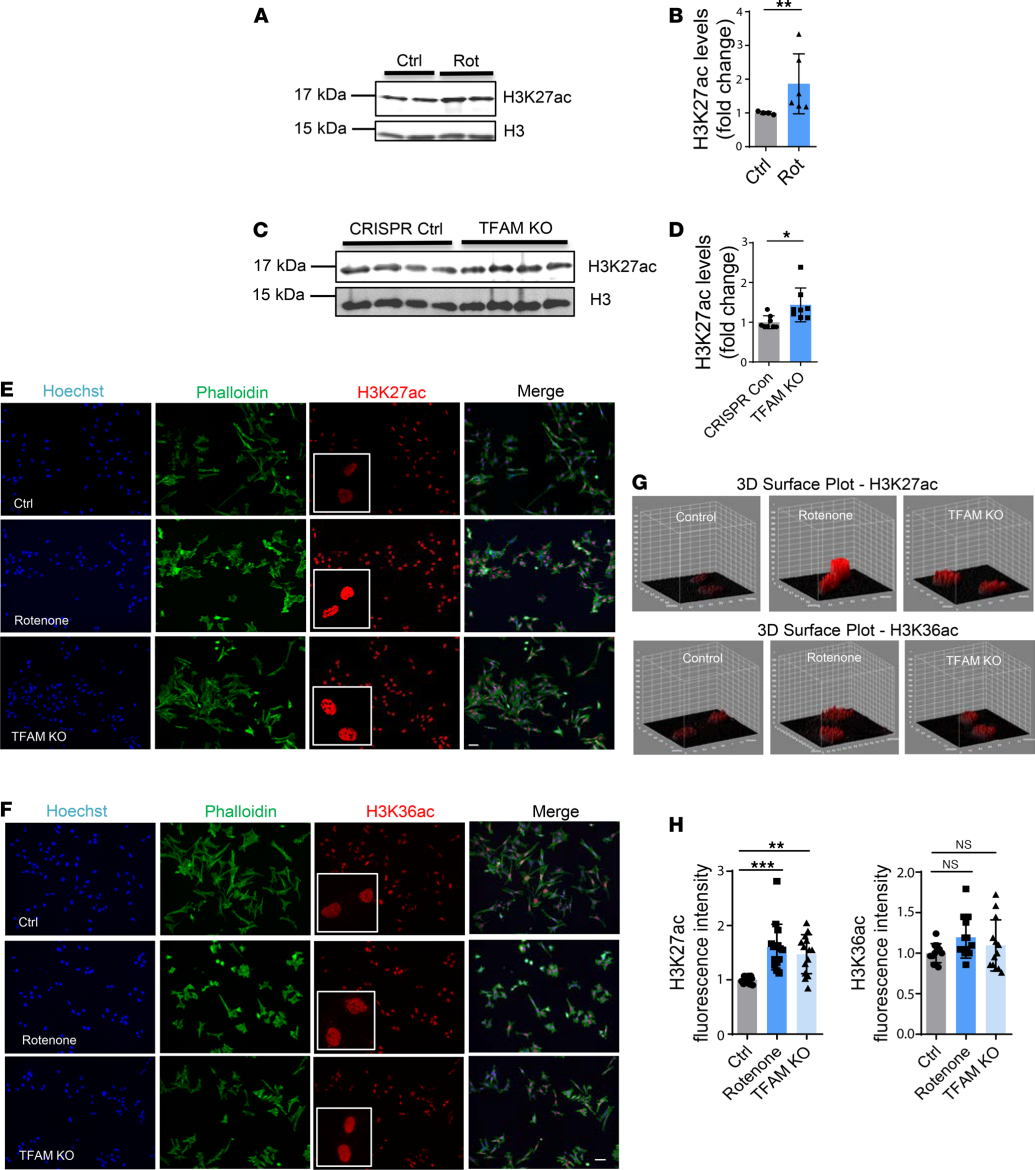
H3K27ac functions as the key acetylation site and responds to mitochondrial dysfunction in both rotenone-exposed and TFAM-KO DAergic neuronal cell models of Parkinson’s disease. (**A**) Representative immunoblot and (**B**) quantification for H3K27ac from rotenone (Rot)-treated N27 cells. Mann-Whitney test (*n* = 4–6). (**C**) Representative immunoblots for H3K27ac and (**D**) quantification from TFAM-KO N27 cells. Unpaired Student’s *t* test followed by Welch’s correction (*n* = 8). In Western blots, 5 T175 flasks of cells were harvested for each sample. (**E**) ICC for H3K27ac and (**F**) H3K36ac from rotenone-treated and TFAM-KO N27 cells, with the nucleus stained with Hoechst (blue) and actin filaments with phalloidin (green). Independent experiments were repeated at least 4 times. Scale bar: 50 μm. (**G**) 3D surface plot analysis of H3K27ac and H3K36ac in rotenone-treated and TFAM-KO N27s. (**H**) Keyence BZ-X800 analysis of fluorescence intensity of H3K27ac and H3K36ac showing mean ± SEM; 1-way ANOVA followed by Tukey’s post hoc test. Ctrl, control; NS, not significant. *P ≤ 0.05; **P < 0.01; ***P < 0.001.

To validate the proteome-wide Kac results in histone H3, we probed rotenone-treated N27 cells for H3K27ac by Western blotting. We used rabbit polyclonal antibodies that recognize the same epitopes within the internal region of H3K27ac in AcetylScan. Immunoblot analysis revealed that H3K27ac increased significantly in rotenone-exposed and mitochondria-impaired N27 cells (Figure 1, A–D), whereas H3K36ac showed minimal accumulation (data not shown). To further establish the elevated H3K27ac response to mitochondrial dysfunction, we added a complementary cellular model with a mitochondrial genetic mutation for independent confirmation. Conditionally knocking out transcription factor A, mitochondrial (TFAM), which normally functions in genomic transcription regulation and controls mitochondrial biogenesis (23), in the nigral DAergic cells of mice produces mitochondrial dysfunction that leads progressively to a suite of neural and behavioral deficits recapitulating PD (24). Using a CRISPR/Cas9 gene-editing method, we generated a stable TFAM-KO N27 cell line. Using a similar set of analyses as described above (Supplemental Figure 2), we also confirmed mitochondrial dysfunction in the TFAM-KO DAergic cell model (Supplemental Figure 3) and a more than 90% loss of TFAM protein through immunoblot (Supplemental Figure 4). In immunocytochemistry, both rotenone-exposed and TFAM-KO cells showed significantly elevated signal intensities of H3K27ac in the nucleus (Figure 1, E, G, and H; red) but the H3K36ac signal was much less affected (Figure 1, F–H), indicating that the H3K27 site is more sensitive to hyperacetylation than the H3K36 site during mitochondrial dysfunction.

Collectively, these findings suggest that acetylation of the H3K27 site, and not that of H3K36, is the primary response to mitochondrial dysfunction in DAergic neuronal cells.

### Rotenone-exposed and TFAM-KO N27 DAergic neuronal cells share similar transcriptomic alterations.

Having established that mitochondrial dysfunction leads to significant changes in Kac profiles, we next determined its consequence for the transcriptome by performing RNA-Seq analyses (3 biological replicates) on both rotenone-treated and TFAM-KO N27 cells. Principle component analysis and hierarchical clustering segregated the transcriptomic profiles of normal N27 cells from rotenone-exposed or TFAM-KO N27 cells (Supplemental Figure 5, A and B).

**Figure 2:**
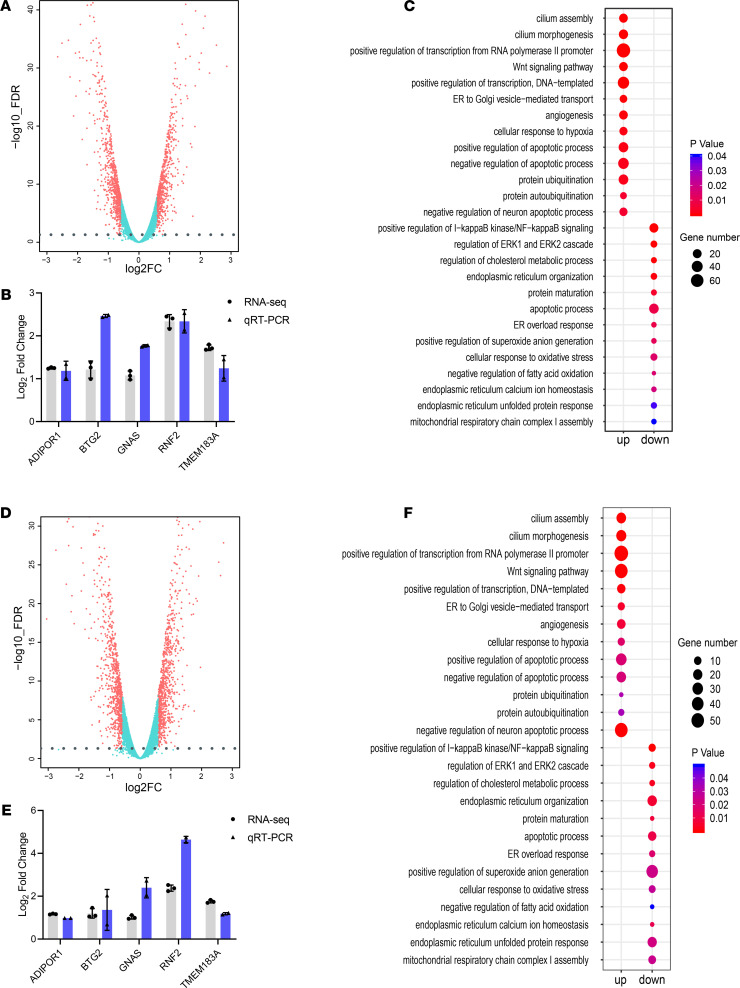
Transcriptomic alterations and common differentially expressed genes in rotenone-treated and TFAM-KO N27 cells. (**A**) Volcano plot showing 644 genes upregulated (right) and 767 genes downregulated (left) upon rotenone treatment. (**B**) RT-qPCR validation of multiple differentially expressed genes (DEGs) upon rotenone treatment. (**C**) GO analysis revealing the top 12 biological processes enriched by upregulated (left) and downregulated (right) genes upon rotenone treatment. (**D**) Volcano plot showing 813 genes upregulated (right) and 862 genes downregulated (left) by TFAM KO. (**E**) RT-qPCR validation of multiple DEGs upon TFAM KO. (**F**) GO analysis revealing the top 12 biological processes enriched by TFAM KO.

We observed that rotenone exposure upregulated 644 genes and downregulated 767 genes (Figure 2A), based on an FDR cutoff of 0.05 (FDR ≤ 0.05) and a greater than 1.5-fold change (log_2_FC ≥ 0.585) in differential expression analysis. To determine how the identified differentially expressed genes (DEGs) relate to relevant PD pathways, we performed Gene Ontology (GO) enrichment analysis for biological processes. We further validated our RNA-Seq results with quantitative reverse transcription PCR (RT-qPCR) analyses for 5 selected genes (*Adiport1*, *Btg2*, *Gnas*, *Rnf2*, and *Tmem183a*) (Figure 2B), which are related either to mitochondrial or neuronal functions. Rotenone-induced DEGs were mainly enriched in the biological processes related to the Wnt signaling pathway, NF-κB translocation, transcription regulation, cell apoptosis, endoplasmic reticulum protein quality control, and cellular stress responses (Figure 2C).

In the TFAM-KO N27 cell model, TFAM KO upregulated 813 genes and downregulated 862 genes based on the same criteria applied to rotenone-treated N27 cells (Figure 2D). We also validated our RNA-Seq results from TFAM-KO N27 cells with the 5 genes selected above for RT-qPCR assay and confirmed their expression changes (Figure 2E). GO enrichment analysis revealed that the TFAM-KO–induced DEGs were enriched in biological processes similar to those observed with rotenone-induced DEGs (Figure 2F).

**Figure 3:**
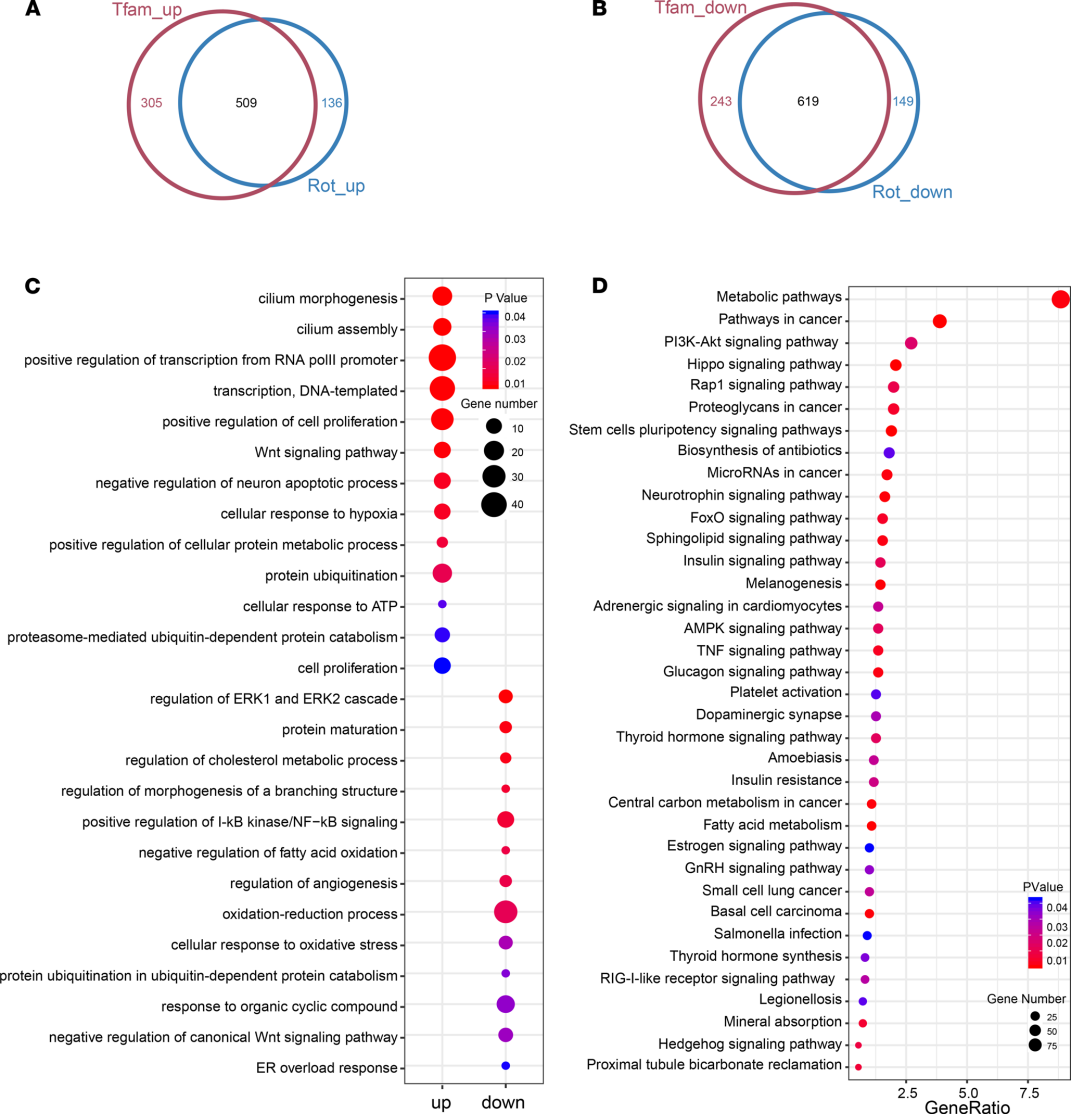
Rotenone-exposed and TFAM-KO N27 DAergic neuronal cells share similar transcriptomic alterations. Venn diagrams highlighting (**A**) upregulated and (**B**) downregulated DEGs shared between rotenone-treated and TFAM-KO N27s. (**C**) GO analysis revealing the top 12 biological processes enriched by upregulated genes (left) and downregulated genes (right) overlapping between rotenone treatment and TFAM KO. (**D**) KEGG pathway analysis identifying the most significant pathways for common, enriched DEGs.

With a large number of genes perturbed in both models, we characterized the extent to which rotenone exposure and TFAM KO shared effects on transcription. The data revealed that most the of up- or downregulated genes were shared in both cell models, including 509 out of 645 rotenone- and 814 TFAM-KO–upregulated genes (Figure 3A) and 619 out of 768 rotenone- and 862 TFAM-KO–downregulated genes (Figure 3B); detailed annotation and fold-change values for these DEGs are summarized in Supplemental Table 1. Our RNA-Seq and RT-qPCR results above agree in both the direction and magnitude of fold change for the 5 selected genes (Figure 2, B and E). Together, these results indicate that environmental neurotoxicant exposure and TFAM genetic mutation induce similar transcriptomic alterations linked to specific biological functions.

Further GO enrichment analysis showed that these overlapping upregulated genes were mainly enriched in the biological processes related to cilium morphogenesis, transcription, Wnt signaling, neuron apoptosis, and protein catabolic processes. Overlapping downregulated genes were largely enriched in the biological processes associated with the ERK1/2 cascade, branching structure, NF-κB signaling, oxidative stress, and protein ubiquitination (Figure 3C).

In addition, Kyoto Encyclopedia of Genes and Genomes (KEGG) pathway annotation was performed to explore the function of the parental genes of the overlapping DEGs from which multiple pathways were identified (Figure 3D). With many DEGs involved, the metabolic pathway was not only the most enriched pathway but also the pathway of highest significance. Additional pathways highly relevant to PD included the apoptosis-related PI3K/AKT signaling pathway, the AMPK signaling pathway activated by falling energy levels yet exacerbating cell death (25), and the TNF signaling pathway associated with mediating neurotoxicity. Collectively, these pieces of evidence further highlight our hypothesized mitochondrial dysfunction-metabolite-H3K27ac-transcriptome axis underlying PD pathogenesis.

### H3K27ac ChIP-Seq identification of vulnerable genomic loci upon mitochondrial impairment.

We next identified what epigenomic loci became more vulnerable to H3K27ac modification and what TFs were activated. We did ChIP-Seq analyses in both rotenone-treated N27 and TFAM-KO N27 cells by using our previously characterized anti-H3K27ac Ab (26). Genome-wide H3K27ac signals were analyzed following our established pipeline.

**Figure 4:**
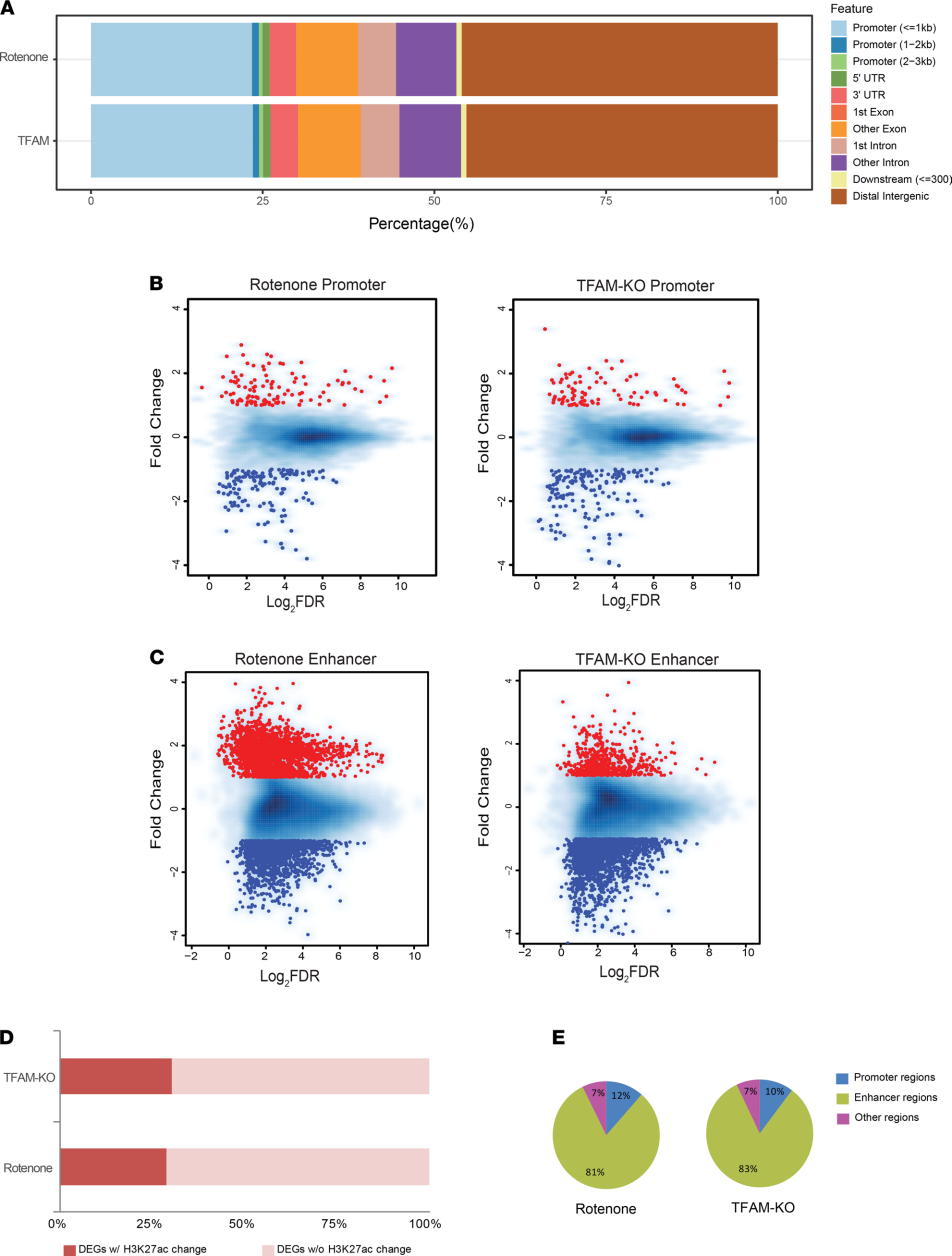
Identification of vulnerable genomic loci binding with H3K27ac in rotenone-treated and TFAM-KO N27 cells. (**A**) Bar chart indicating the similar genomic distribution of H3K27ac sites in 2 models after intragroup normalization. (**B**) Scatter plots showing H3K27ac peaks located at gene promoter regions in rotenone-treated and TFAM-KO N27 neurons when compared with their respective control. (**C**) Scatter plots showing H3K27ac peaks located at enhancer regions in rotenone-treated and TFAM-KO N27 neurons when compared with their respective control. Each point represents a peak and most points cluster in the middle. The dots indicate upregulated (red) and downregulated (blue) H3K27ac peaks. (**D**) Bar plot showing the proportion of differentially expressed genes (DEGs) with a consistent change in H3K27ac enrichment in both models. (**E**) Pie charts indicating the genomic features of those altered H3K27ac peaks in both models.

Hierarchical clustering analysis using affinity score (read count) data showed that H3K27ac had similar binding intensities among all of our samples, its distribution was closely correlated among biological replicates, and was discernibly redistributed upon either rotenone exposure or TFAM KO (Supplemental Figure 5, C and D). Annotation for these identified H3K27ac peaks reveals that approximately 25% of these peaks intersected with annotated genes or their proximal promoters (hereafter defined as gene promoter regions located within 3 kb upstream and downstream of the transcription start sites), although most of these peaks (approximately 57%) corresponded to noncoding intragenic and distal intergenic regions (Figure 4A and Supplemental Figure 5, E and F). The genomic distribution of H3K27ac did not differ significantly between rotenone-treated and TFAM-KO cells.

When examining whether mitochondrial impairment triggers H3K27ac modification, we found that 11.5% of H3K27ac peaks were significantly changed after rotenone treatment and 8.0% of H3K27ac peaks were significantly changed after TFAM-KO cell treatment (Supplemental Figure 5G). By looking into the genomic distribution of these altered H3K27ac enrichments, 2.79% and 2.82% of H3K27ac peaks at annotated genes and their gene promoter regions were significantly changed after rotenone and TFAM-KO cell treatment, respectively (Figures 4B and Supplemental Figure 5G). Using H3K27ac as an enhancer-specific histone modification mark, we found that 16.6% and 10.9% of H3K27ac peaks at distal intergenic regions and noncoding intragenic regions (hereafter called enhancer regions) were significantly changed after rotenone and TFAM-KO cell treatment, respectively (Figure 4C and Supplemental Figure 5G). These results indicate that large numbers of enhancers defined by the H3K27ac mark were sensitive to mitochondrial impairment, which has implications for enhancer-specific gene regulation related to PD pathogenesis.

**Figure 5:**
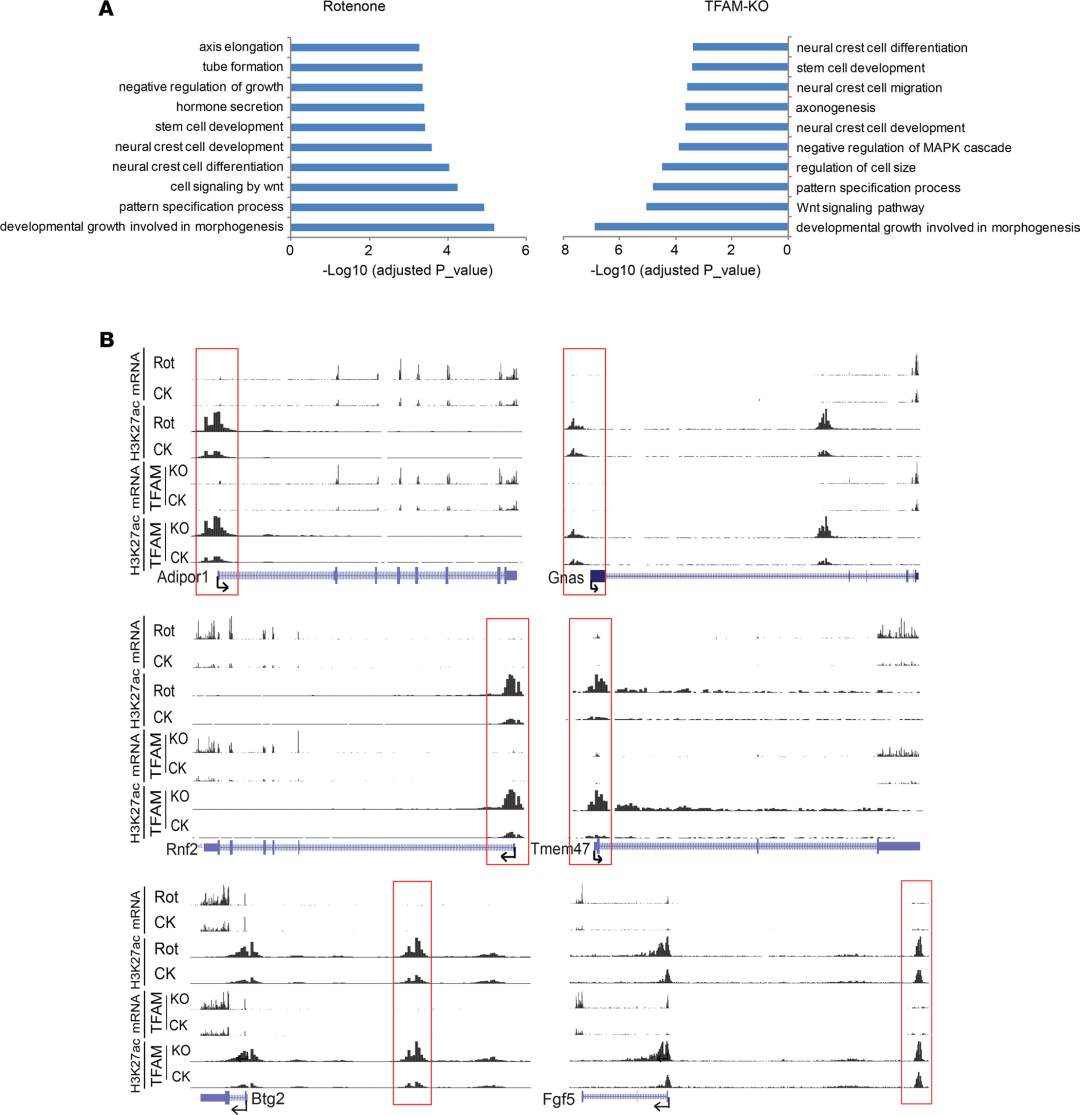
Identification of H3K27ac-specific enhancers in rotenone-treated and TFAM-KO N27 cells. (**A**) GO biological processes associated with differentially expressed genes (DEGs) of differentially enriched H3K27ac in both models. (**B**) Representative distribution of reads obtained by ChIP-Seq and RNA-Seq in selected gene loci related to mitochondrial dysfunction and neural function in N27 cells. Red rectangular boxes indicate promoter or enhancer regions. The distribution of H3K27ac was normalized to input and library dimension. Uniform scales are used for H3K27ac and for mRNA. CK, vehicle control; TFAM KO, TFAM KO by CRISPR/Cas 9; TFAM CK, TFAM CRISPR control.

We next determined whether H3K27ac modifications were responsible for any gene transcription alterations (13, 26). By integrating the RNA-Seq data, we found that approximately 30% of the genes modulated by either rotenone exposure or TFAM-KO cells had undergone altered H3K27ac enrichment (Figure 4D). Among those altered H3K27ac signals, 10%–12% were located at promoter regions, whereas more than 80% were located at enhancer regions (Figure 4E). GO analysis revealed that a significant number of the DEGs associated with the H3K27ac modification in both models are implicated in biological processes such as organ development, cell proliferation and differentiation, nervous system development, and Wnt signaling transduction (Figure 5A). Thus, H3K27ac provides an epigenetic signature that distinguishes genes regulated in neural dysfunction induced by mitochondrial impairment, with consistent alteration of transcription and H3K27ac enrichment occurring at promoter and/or enhancer regions, such as *Adipor1*, *Gnas*, *Rnf2*, *Tmem47*, *Btg2*, and *Fgf5* (Figure 5B).

### Characterization of TF-binding motifs specific to mitochondrial impairment.

Given that multiple genomic regions, especially enhancer regions, were influenced by mitochondrial impairment, we further searched for the presence of TF-binding motifs within these genetic elements to identify TFs that potentially interact with aberrant H3K27ac caused by mitochondrial impairment.

**Figure 6:**
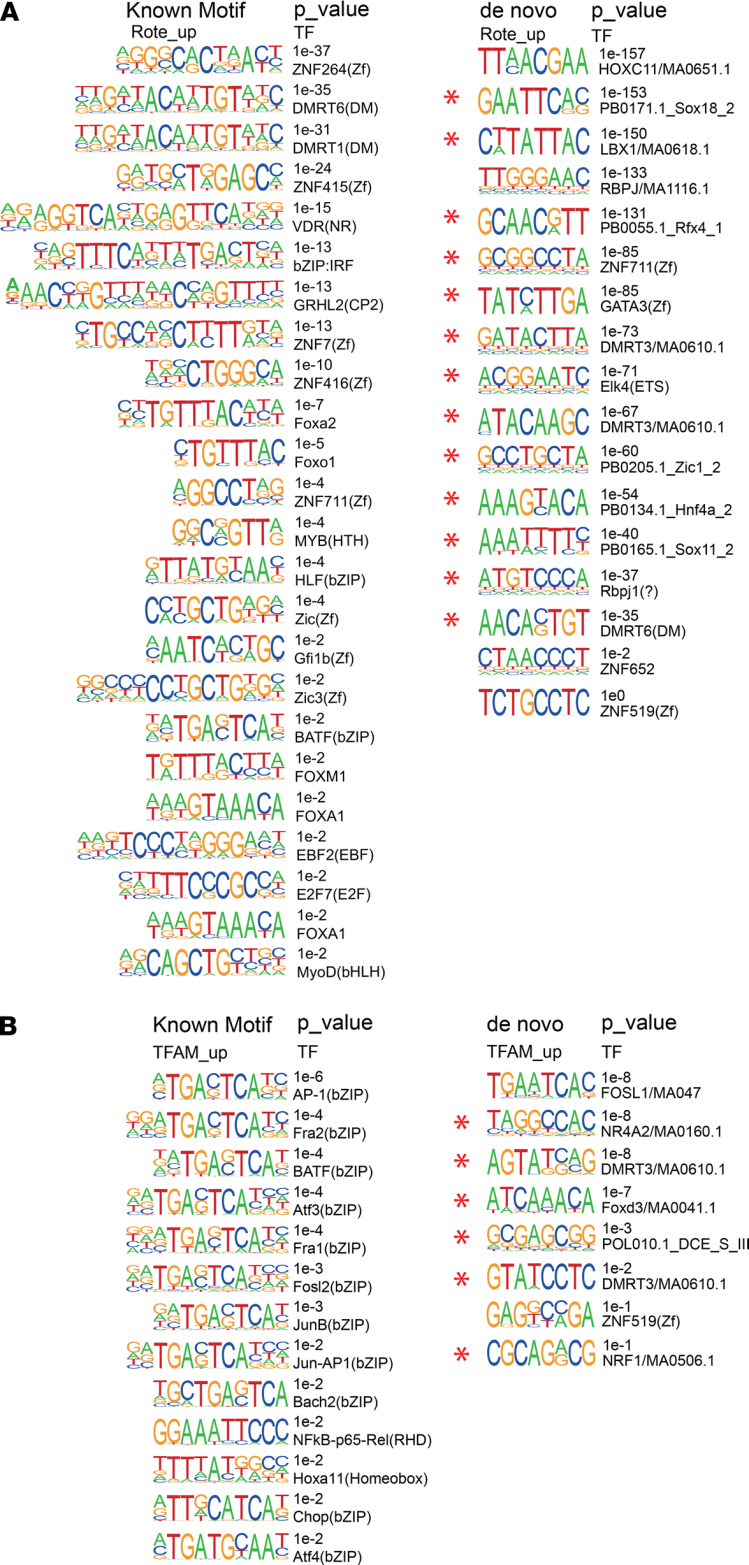
H3K27ac-specific motif analysis in rotenone-treated and TFAM-KO N27 cells. (**A**) Transcriptional factor (TF)–binding motifs enriched in rotenone-affected (Rote-affected) enhancers and (**B**) TFAM-KO–affected enhancers. The top enriched known motifs and corresponding TFs appear in the left panel, whereas the de novo motifs appear in the right panel with their best-known TF matches.

Since we are particularly interested in the regions bound by elevated H3K27ac, we applied the HOMER tool to scan for TF-binding motifs within upregulated H3K27ac peaks at enhancer regions. Meanwhile, for the background control, we also analyzed the TF-binding motifs within randomly selected H3K27ac peaks that were not changed in both models (Supplemental Table 2). Most of the enriched motifs in rotenone-upregulated H3K27ac peaks corresponded to TFs with a characteristic ZF (zinc finger DNA-binding) domain or DM (zinc finger-like DNA-binding) domain (Figure 6A). ZF proteins like ZNF264 contribute to coordinated gene expression changes during brain aging (27) and ZNF711 is reportedly associated with mental retardation and cognitive disability (28). The DM family of TFs such as DMRT1 and DMRT3 are involved in sexual development (29) and movement control (30, 31). In contrast, bZIP motifs were more enriched in TFAM-KO–upregulated H3K27ac peaks (Figure 6B). The family of bZIP TFs has been assigned important roles in cancer development, hormone synthesis, and reproductive functions (32–34).

To further explore the unknown motif landscape of enhancers, we applied de novo motif discovery to upregulated enhancers caused by rotenone and TFAM-KO exposure (Figure 6, A and B). Although these potential novel motifs were lacking perfect database matches, HOMER provided similar known motifs that most closely matched the de novo motifs. The most enriched motif in rotenone-upregulated enhancers was related to the homeobox family that plays an important role in morphogenesis in all multicellular organisms (35). The other enriched motif in rotenone-upregulated enhancers was closely matched with the recombination signal-binding protein for immunoglobulin kappa J region (RBPJ) TF, which reportedly controls the notch signaling pathway by recruiting histone deacetylase (HDAC) or histone acetyltransferase (HAT) (36). The most enriched motif in TFAM-KO–upregulated enhancers was matched with FOLS1 (Fos-like 1), a component of AP-1 TF complexes that contributes to the regulation of placental development (37). By identifying the known and unknown TF motifs in neurodegeneration driven by mitochondrial dysfunction, our investigations reveal the intricate connections between epigenetic and transcriptional regulation, providing insight into understanding PD pathogenesis.

### Mitochondrial dysfunction–induced H3K27ac mediates mitonuclear communication and possibly the loss of DAergic midbrain neurons.

Bidirectional mitonuclear communication maintains cellular homeostasis and cellular adaptation to environmental stress. Retrograde response signaling from mitochondria to the nucleus adjusts gene expression through epigenomic modifications (19, 38–42). Having shown that mitochondrial dysfunction–induced H3K27ac mediates retrograde responses and subsequently alters gene expression in vitro, we then extended these findings ex vivo to the acutely rotenone–exposed midbrain slice model as well as in vivo to the more progressively neurodegenerative MP Tg mouse model. Immunoblotting showed H3K27ac significantly increasing in rotenone-exposed slice cultures of the mouse substantia nigra (SN), but not in the striatum, revealing a brain region–specific response (Supplemental Figure 6, A–C) consistent with our in vitro findings.

**Figure 7:**
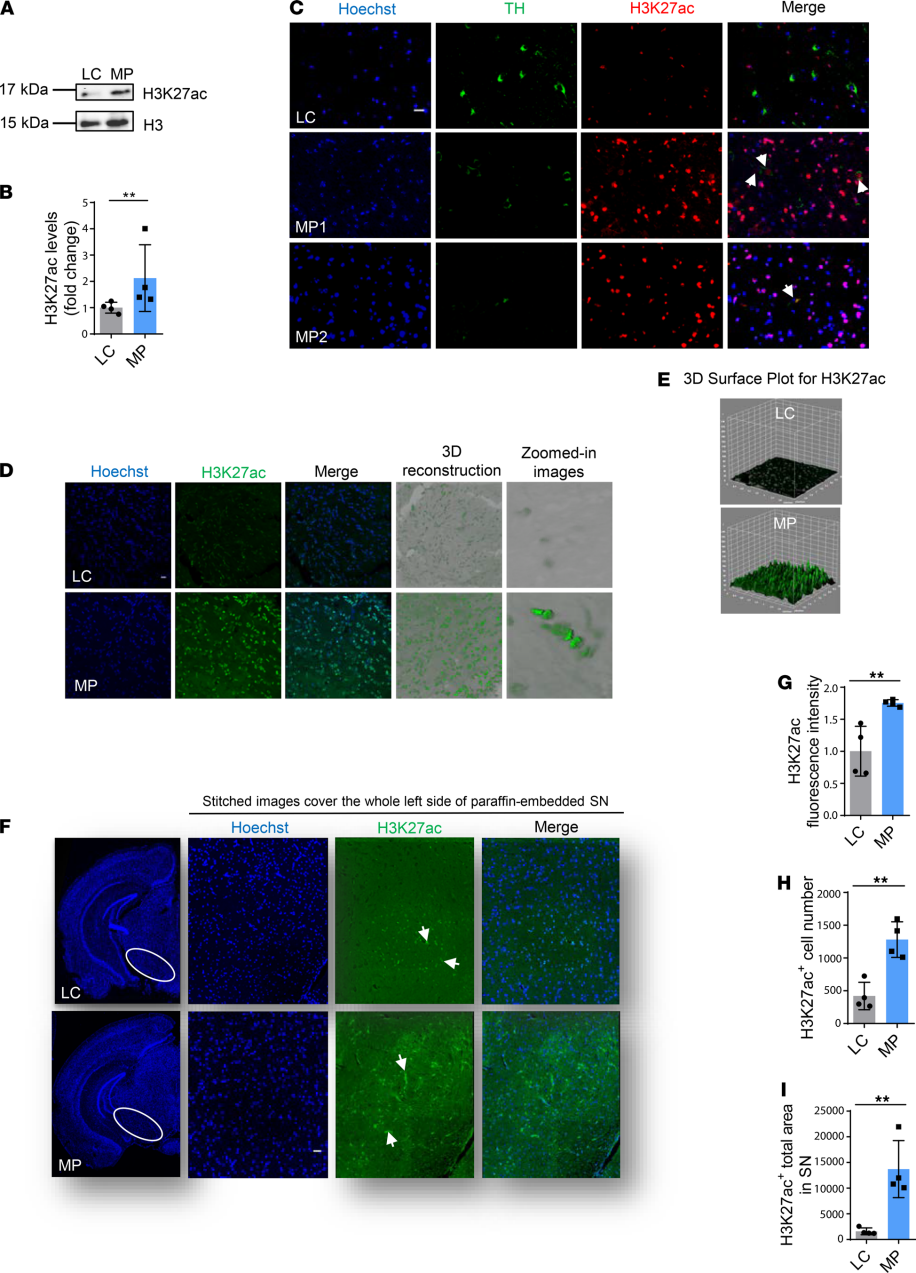
Elevated H3K27ac in substantia nigra of MitoPark mice. (**A**) Representative immunoblots and (**B**) their quantification from MitoPark (MP) and littermate control (LC) mice. Mann-Whitney test (*n* = 4). (**C**) Zoomed-in images (scale bar: 5 μm) of 7-μm-thick paraffin-embedded sections (shown in Supplemental Figure 6G) with H3K27ac in red, nucleus in blue, and TH in green. MP1 shows not only colocalization of TH and H3K27ac but also several degrading TH neurons, whereas MP2 reveals degraded TH neurons and minimal colocalization. Arrows denote colocalization of H3K27ac and TH. (**D**) Confocal images of 30-μm free-floating sections from same-aged mice with H3K27ac in green and nucleus in blue. Scale bar: 5 μm. (**E**) 3D surface plot analysis for H3K27ac. (**F**) Images stitched together from 150 images (original magnification, ×60) showing the entire region of the substantia nigra (SN) (H3K27ac, green; nucleus, blue). Arrows denote H3K27ac. Scale bar: 400 μm. (**G**) Quantified fluorescence intensity for H3K27ac. (**H**) Count of cells stained with H3K27ac. (**I**) Area of SN with cells stained with H3K27ac. Each data point is the average of 3 replicates. For IHC of substantia nigra, *n* = 11–12. Independent experiments were repeated 4 times. Bar graphs show mean ± SEM; unpaired 2-tailed Student’s *t* test. NS, not significant. **P < 0.01.

Next, we lysed the SN and striatum of 16- to 20-week-old MP and littermate control (LC) mice to probe with H3K27ac. Again, immunoblotting results show significantly increased H3K27ac in the MP SN but not in the striatum, suggesting a possible link between H3K27ac and nigral DAergic neurodegeneration (Supplemental Figure 6, D–F, and Figure 7, A and B). To further confirm the colocalization of H3K27ac and degenerating DAergic neurons, we performed IHC using 7-μm paraffin-embedded sections to probe H3K27ac (green) and the DAergic neuron biomarker tyrosine hydroxylase (TH; red) in the SN of 16- to 20-week-old MP and LC mice. In contrast to LCs, TH mostly degraded in MPs concomitant with the colocalization and significant accumulation of H3K27ac (Supplemental Figure 6G and Figure 7C). Locomotor and RotaRod coordination tests confirmed PD-like neurobehavioral deficits in these MP mice (Supplemental Figure 7). This demonstrates the colocalization of H3K27ac and dying TH neurons, suggesting that H3K27ac contributes to the degeneration of DAergic neurons.

To further quantify H3K27ac accumulation, 30-μm free-floating nigral sections from 16- to 20-week-old MP and LC mice were stained, and 3D and *Z*-stack images were captured by confocal imaging. Substantial H3K27ac (green) accumulation in the MP SN is evidenced by 3D surface plot analysis (Figure 7, D and E). For quantifying nigral H3K27ac, high-resolution images were stitched together to cover the entire SN region (Figure 7F). Quantification of H3K27ac fluorescence intensity (Figure 7G), cell count (Figure 7H), and cell area (Figure 7I) via automated BZ-X800 analyzer confirmed significant H3K27ac accumulation in the SN of mitochondria-impaired MPs, supporting our in vitro and ex vivo data.

Collectively, these data demonstrate that mitochondrial impairment induced H3K27ac accumulation and bookmark epigenetic changes in the mouse brain, which may contribute to neurodegeneration.

### H3K27 hyperacetylation in the SN of human PD brains.

**Figure 8:**
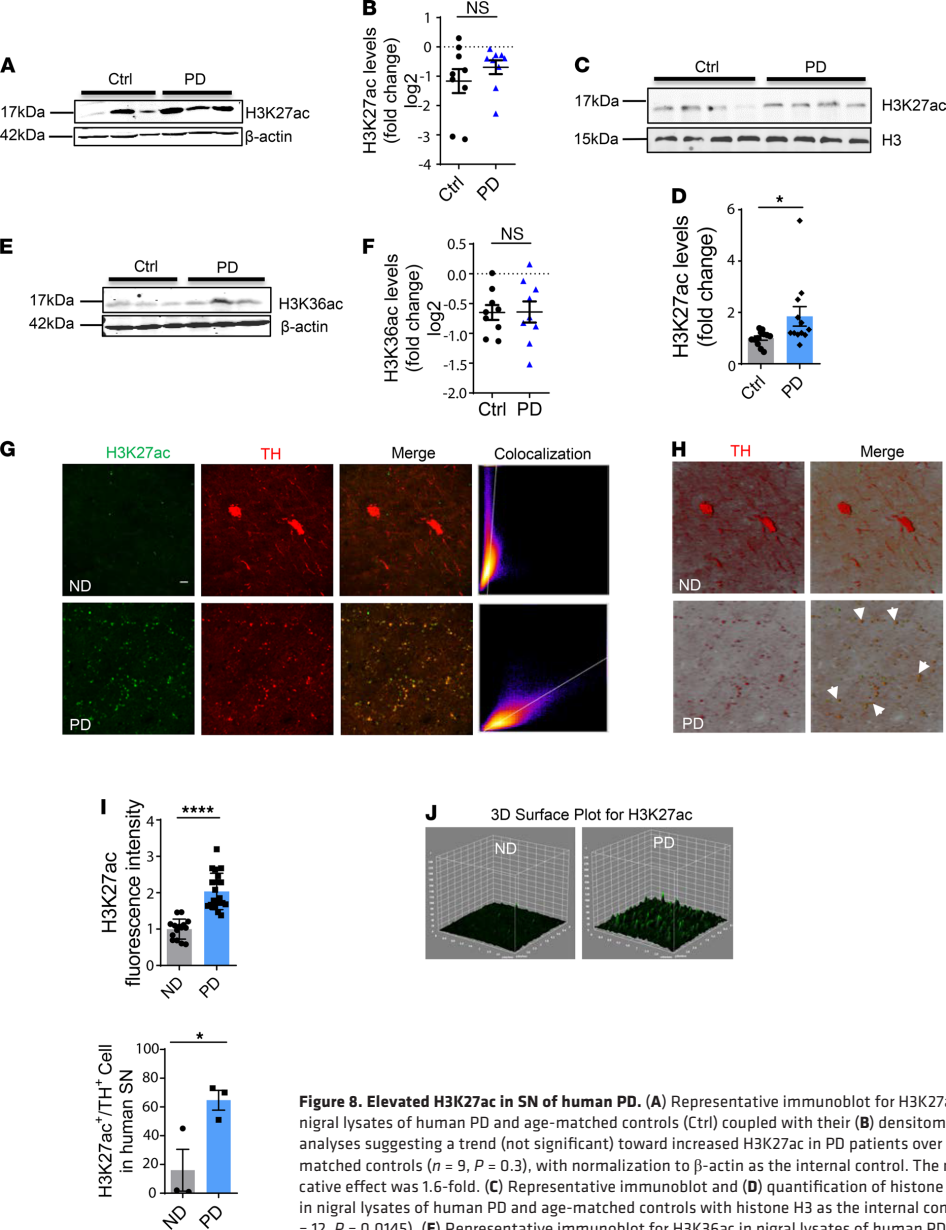
Elevated H3K27ac in SN of human PD. (**A**) Representative immunoblot for H3K27ac in nigral lysates of human PD and age-matched controls (Ctrl) coupled with their (**B**) densitometric analyses suggesting a trend (not significant) toward increased H3K27ac in PD patients over age-matched controls (*n* = 9, *P* = 0.3), with normalization to β-actin as the internal control. The multiplicative effect was 1.6-fold. (**C**) Representative immunoblot and (**D**) quantification of histone extracts in nigral lysates of human PD and age-matched controls with histone H3 as the internal control (*n* = 12, *P* = 0.0145). (**E**) Representative immunoblot for H3K36ac in nigral lysates of human PD brains and age-matched controls (*n* = 9) coupled with their (**F**) densitometric analyses revealing no difference in H3K36ac between human PD and age-matched controls. Data in **B** and **F** are represented on a logarithmic scale for outlier resistance. (**G**) IHC in SN sections of human PD and age-matched nondisease (ND) controls (*n* = 3) captured by confocal imaging showing a high level of colocalization (orange) between H3K27ac (green) and TH (red) in PD, based on (**H**) colocalization plot analysis and 3D images; arrows denote colocalization. Scale bar: 10 μm (**G** and **H**). (**I**) Quantified fluorescence intensity for H3K27ac and count of cells with colocalization in 5 randomly selected regions of each human SN section. (**J**) 3D surface plot analysis for H3K27ac. Bar graphs show mean ± SEM; unpaired 2-tailed Student’s *t* test. NS, not significant; *P ≤ 0.05; ****P < 0.0001.

Next, we explored the human relevance of H3K27ac to highlight the significance of our in vitro and in vivo findings. To this end, postmortem SNs of PD patients and age-matched controls were lysed and immunoblotted with the antibody against H3K27ac. After observing increased total H3 in PD patients, we used both whole SN lysates and histone extracts. H3K27ac in whole SN lysates of human PD patients was 1.6 times higher than that in age-matched controls, suggesting that H3K27ac had been stimulated in PD patients (Figure 8, A and B). Immunoblotting of nuclear histone extracts from more SN samples of PD patients and age-matched controls against H3K27ac validates the significant increase of H3K27ac accumulation in the SN of PD patients (Figure 8, C and D). To rule out the possibility that H3K36ac instead is the critical player, we immunoblotted the SN of PD patients and age-matched controls with an H3K36ac antibody (Figure 8, E and F). No obvious difference in nigral H3K36ac emerged between PD and age-matched controls (*P* value = 1.0), further confirming our findings in vitro that H3K36ac does not function as a critical player in response to mitochondrial dysfunction (Figure 1, F and G).

To further corroborate the establishment of the H3K27ac epigenetic mark in the SN of human PD patients and characterize the possible relationship between H3K27ac and DAergic neurodegeneration, we immunostained human nigral sections for H3K27ac (green) and TH (red). IHC analysis showed (a) significantly increased H3K27ac in nigral human PD patients compared with age-matched controls, consistent with Western blot results; (b) a massive degradation of TH neurons in PD patients indicative of neurodegeneration; and (c) more than 50% colocalization (orange) of H3K27ac and DAergic neurons in the SN of PD patients (Figure 8, G–J).

Collectively, our human SN data demonstrate strong colocalization of H3K27ac and TH neurons, adding clinical relevance to our findings from experimental models of PD.

### H3K27ac increases TMEM47 expression in the SN of MP mice and postmortem human PD brains.

**Figure 9:**
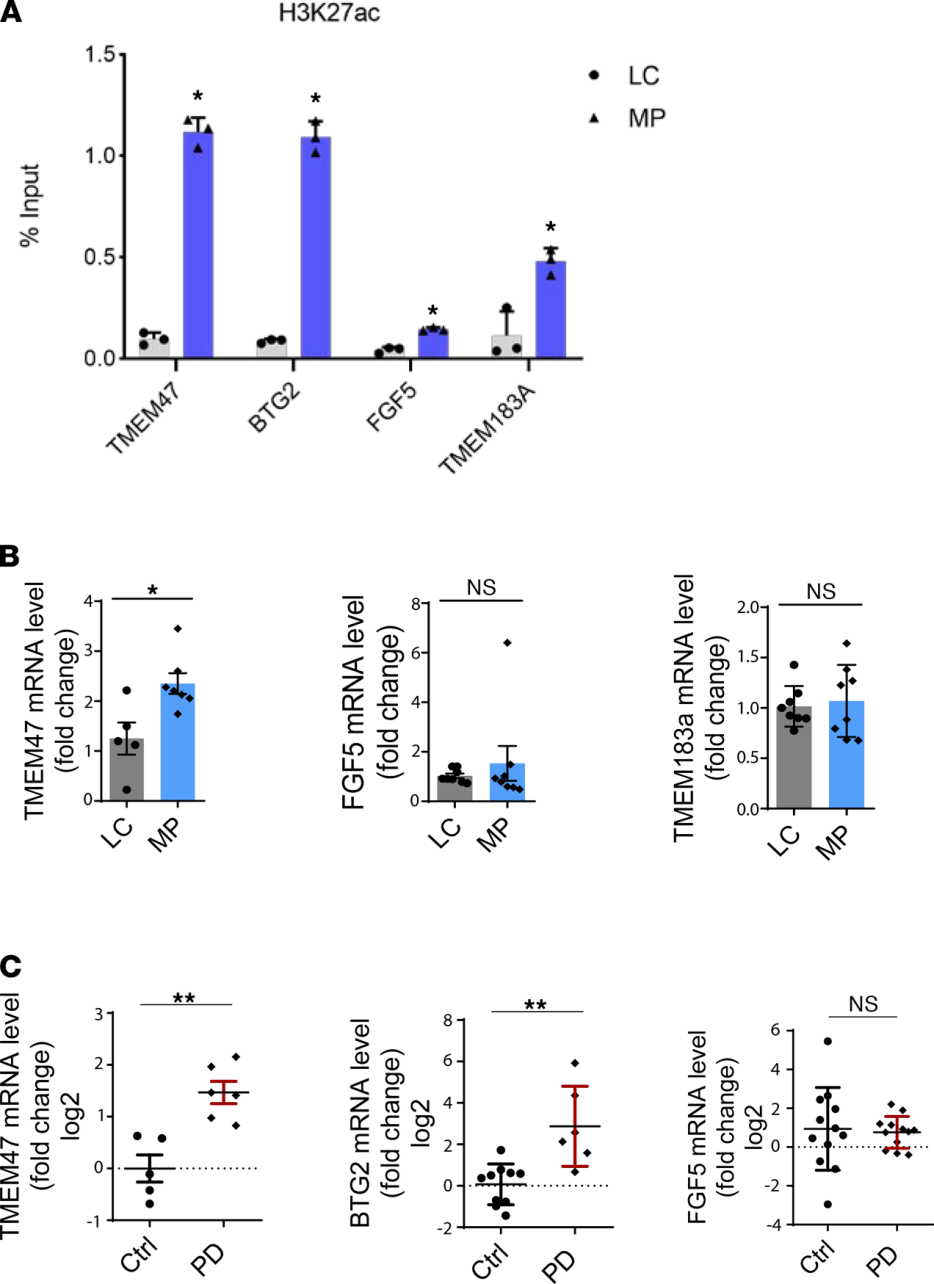
Elevated mRNA levels of selected genes in SN of MP and human PD. (**A**) ChIP-qPCR validation of higher enrichment of H3K27ac at multiple gene promoters using littermate controls (LCs) and MP mice (*n* = 4). (**B**) RT-qPCR of selected genes from integrated RNA-Seq and ChIP-Seq data in SN of LC and MP mice and (**C**) postmortem SN from age-matched human control and PD subjects with *n* = 3 (2 technical replicates). Bar graphs show mean ± SEM of unpaired 2-tailed Student *t* tests. NS, not significant; *P ≤ 0.05; **P < 0.01.

To independently confirm our in vitro findings of H3K27ac at genomic loci, we performed ChIP-qPCR analyses in vivo. The SN of randomly chosen 16- to 17-week-old MP and age-matched LC mice were dissected for ChIP-qPCR. MPs exhibited significantly higher enrichment of the genes *Tmem47*, *Btg2*, *Fgf5*, and *Tmem183a* (Figure 9A), which agreed with our ChIP-Seq results in N27 DAergic neuronal cells.

To further examine the effect of H3K27ac on gene expression, several upregulated candidate genes were selected from our integrated analyses of RNA-Seq and ChIP-Seq. Our qPCR analyses of the SN region confirmed enhanced mRNA levels of *Tmem47* in MP mice (Figure 9B) and human PD brains (Figure 9C), which also confirms our H3K27ac reads at genomic loci for neuronal models (Figure 5B) and ChIP-qPCR data (Figure 9A). Consistent with MP mice and human PD brains, midbrain slice cultures show increased *Tmem47* mRNA levels (Supplemental Figure 8). As a transmembrane protein, *Tmem47* (previously known as *Tm4sf10* or *Bcmp1*) reportedly regulates Fyn kinase (43). Our previous publication identified a proapoptotic Fyn/PKCδ signaling axis that contributes to pesticide-induced cell death in DAergic neuronal cells (44–46). However, the actual function of *TMEM47* in human PD remains unclear. Increased transcription levels of *BTG2* also occurred in human PD brains (Figure 9C), in agreement with the SN region of midbrain slice cultures (Supplemental Figure 8), enhancer data (Figure 5B), and ChIP-qPCR data (Figure 9A). *Btg2* targets p53, which regulates cell cycle progression and mitochondrial depolarization (Supplemental Figure 2A and Supplemental Figure 3A) (47). *Btg2* expression strengthens neurons against mitochondrial permeability transitions and promotes neuronal survival under cellular stress (48). *FGF5* and *TMEM183a* expression was not altered in the SN of MP mice and human PD samples, as measured by their mRNA levels (Figure 9, B and C). Furthermore, transcription levels of *Fgf5*, *Ptgs2*, *Rnf2*, *Btg2*, *Tmem183a*, and *Tmem47* did not increase in the MP striatum (Supplemental Figure 9), as expected.

## Discussion

Emerging evidence supports the significance of histone modification in the maintenance of a healthy brain and as a potentially useful therapy to correct transcriptional dysfunction in PD (49, 50). Dating back to the work of Song et al. (9) on pesticide-induced global histone modification in cell culture models, our lab has been exploring the mechanisms involved in histone hyperacetylation to unravel the complex multifactorial etiology of PD. This study is the first to our knowledge to systematically address the histone hyperacetylation mechanism together with a genome-wide profile of H3K27ac induced by mitochondrial dysfunction in cell culture and animal models of both environmentally and TFAM genetic variant–linked PD.

To tackle the challenge of the low abundance and substoichiometric nature of potential changes (51–53), we used an optimized AcetylScan method that combines antibody enrichment of posttranslational modification–containing (PTM-containing) peptides with liquid chromatography and tandem mass spectrometry (LC-MS/MS) as part of our in-depth acetylome study (54). In our AcetylScan data, H3K27ac and H3K36ac tend to acetylate together. These 2 sites may act cooperatively to prepare chromatin for transcriptional activation, though the signal peak seems primarily attributable to H3K27ac. Our previous data also showed decreased HDACs upon neurotoxicant exposure (9). Therefore, further investigations on the HDAC/HAT imbalance from the perspective of protein degradation may provide additional insight into the mechanisms underlying mitochondrial dysfunction–induced H3K27ac dysregulation.

Comparing rotenone-treated and TFAM-KO N27 neuronal cell models further revealed that an environmental neurotoxicant and TFAM genetic variant can induce similar transcriptomic changes that converge on shared gene expression changes associated with specific functions. We provide evidence for shared functional processes and pathways among DEGs in these 2 models of mitochondrial dysfunction. Among the DEGs in these 2 cell models, strong enrichment occurred in the biological processes related to the Wnt signaling pathway, ciliary functions, estradiol responses, and neuronal apoptosis, ERK1/2 cascade, and NF-κB translocation, all of which have previously been associated with PD dysfunctions (55–57). For example, impaired Wnt signaling in DAergic neurons has been associated with pathogenesis in a rotenone-triggered PD model (57). Loss of cilia will decrease the ability of cholinergic neurons to respond to a sonic hedgehog signal that triggers neuroprotective signaling toward DAergic circuits (55). Persistent activation of neuronal ERK1/2 and NF-κB translocation has been suggested to mediate neuronal apoptosis and neuroinflammatory responses in PD (58, 59). It is worth noting that protein catabolic processes are also enriched in both cell models. Specifically, the ubiquitin-dependent proteasome pathway has been repeatedly demonstrated to be associated with a wide range of neurodegenerative diseases (60, 61).

To examine the epigenomic aberrations in mitochondria-impairing PD models, genome-wide H3K27ac profiling by ChIP-Seq was analyzed. Overall, specific H3K27ac signatures were identified at promoters and enhancers acting as regulatory elements associated with mitochondrial impairment. Among the identified differential H3K27ac peaks, less than 3% were located at gene promoter regions in either rotenone-exposed or TFAM-KO N27 cells. Several genomic loci appeared to be consistent alteration targets of promoter-H3K27ac modification and gene expression in both model systems tested, including *AdipoR1*, *Gnas*, *Gnf2*, and *Tmem47*. *AdipoR1* is associated with mitochondrial function and ATP production (62, 63). *Rnf2* gene expression was dysregulated in striatally lesioned brain hemispheres (64). An *Rnf2* deficiency increases autophagy induction (65). The imprinted *Gnas* gene can accelerate neuron death (66–68). *TMEM47* is consistently upregulated in vitro, ex vivo, in vivo (MP), and in the postmortem human SN and has been linked to neuronal development and/or brain tumors (69, 70). Interestingly, it is located on the human X chromosome. We speculate that *TMEM47* might be linked to sex differences in PD, as XY males are more susceptible to developing PD than are XX females (71). Additionally, *TMEM47* is known to regulate Fyn kinase, a major member of the Src kinase family. Fyn kinase regulates NLRP3 inflammasome activation, mediates the endotoxic inflammatory response, and modulates uptake of misfolded α-synuclein (46, 72, 73), thereby contributing to neuroinflammation and α-synuclein aggregation as the 2 important hallmarks of PD pathophysiology. Fyn kinase also mediates the phosphorylation of PKCδ Y311, and once activated, PKCδ induces DAergic neuronal degeneration (44). DMRT3 has been referred to as a “gait keeper,” recognizing its crucial role in controlling gait and the flexibility of locomotor coordination in animals (30, 31). Surprisingly, nothing is known about DMRT3 in PD, despite gait disturbance being a major neurological symptom of the disease. With this key function in mind, DMRT3 in PD etiology will be our major focus in the future. Overall, our study identified multiple key genes associated with mitochondrial dysfunction and neurodegeneration. However, little is known about how these genes contribute functionally to PD pathogenesis. Therefore, further experimentation (e.g., H3K27 acetylation inhibition, site mutation, gene knockdown) would be needed to reveal how gene function is linked to changes in H3K27ac in both toxicant and transgenic PD models.

For promoter-H3K27ac aberrations, we observed relatively more differential H3K27ac peaks at distal intergenic and noncoding intragenic regions upon mitochondrial impairment. This is consistent with previous studies. H3K27ac is considered to be the representative acetylation mark for highlighting active enhancers (74) and is more predictive of enhancers than other histone marks (e.g., H3K4me1, H3K4me2, H3K4me3, and H3K9ac) (75). Enhancers are nondirectional regulatory elements containing TF-binding motifs. Upon binding with TFs, complexes are formed and alter the 3D conformation of chromatin to promote the transcription of target genes located in *cis* (76). Population-scale genetic studies indicate that many sequence variants associated with human diseases reside in enhancers (77, 78). We found that large numbers of H3K27ac-defined enhancers were highly sensitive to the mitochondrial dysfunction resulting from rotenone exposure or TFAM KO. For example, *BTG2* is involved in the regulation of the G_1_/S transition of the cell cycle, as well as cell apoptosis and differentiation (79). *FGF5* is involved in regulating neuron differentiation and survival and probably affects astrocytes (80, 81). Among the TFs highlighted by motif analysis of upregulated H3K27ac peaks, the ZF factors ZNF264 and ZNF711 show a strong genetic association with neurological functions (27, 28). AP-1, another enriched TF, reportedly promotes microglial neuroinflammation and DAergic neuron apoptosis in the SNpc (82). Interestingly, AP-1 has also been identified as a key TF enriched by H3K27ac in the brain tissues of a large cohort of patients with autism spectrum disorder (83). Furthermore, apart from these known motifs, our de novo motif discovery identified potentially novel noncanonical motifs, providing additional insight into TF-binding preferences. These results suggest that mitochondrial dysfunction, whether induced through neurotoxicant exposure or genetic mutation, influences the binding of distal H3K27ac-defined enhancers (regulatory elements), thus affecting the gene expression contributing to the etiology of PD.

In addition to the previously mentioned in vitro data, we report here for the first time to our knowledge the PD-relevant, increased accumulation of H3K27ac in an ex vivo model (rotenone-exposed midbrain slice cultures), in the in vivo MP mouse model (a TFAM conditional KO PD), and in the postmortem human SN. Both complementary mouse models helped link the HAc site to mitochondrial dysfunction in the selective dopaminergic neurodegenerative process. For human nigral section staining, we observed small TH fragments in PD patients that were likely the remnants of degraded TH neurons. It is also plausible that non-DAergic neurons are converted into DAergic neurons upon degeneration of the original DAergic neurons. This is supported by the recent publication showing that DAergic neurons could be generated by directly converting nerve cells without reverting them to a stem cell–like state (84). At the same time, it might also be that some of the large numbers of highly plastic neuroblasts that migrate as chains toward the olfactory bulb along the rostral migratory stream, originating from the subventricular zone of the lateral ventricle, de-chain and become DAergic neurons (85). In parallel, the increased H32K7ac accumulation we documented in the SN of human PD brains is coincidentally supported by the results of a prefrontal cortex study of PD from the Netherlands Brain Bank and the Norwegian ParkWest study (86). Although profiling H3K27ac through genome-wide acetylome mapping, ChIP-Seq, and RNA-Seq in PD models can provide important information on PD pathogenesis, our data are limited to a modest number of human samples. Thus, examining more samples would enable us to further interrogate the functional consequences of PD-associated DEGs.

**Figure 10:**
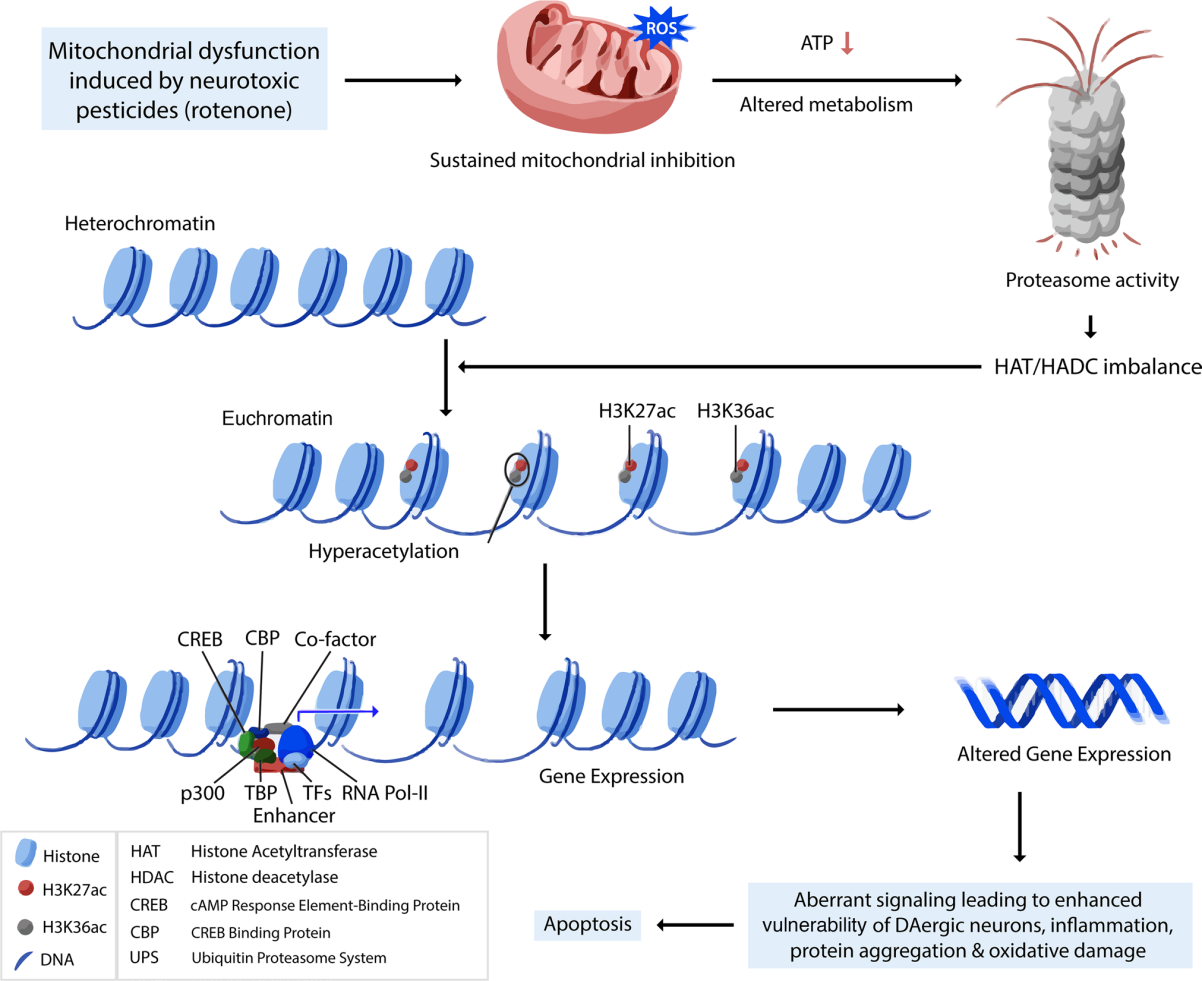
A proposed model for H3K27ac dysregulation in DAergic neurodegeneration. Mitochondrial dysfunction induces epigenetic dysregulation by H3K27 hyperacetylation, which perturbs active enhancers in PD. Mitochondrial deficit results in decreased ATP and altered metabolism. These, together with impaired proteasome activity and HDAC/HAT imbalance, induce histone hyperacetylation on the site of K27, accompanied with K36. H3K27ac dysregulation, a key dysregulation mediator, perturbs the transcriptomic profile and affects gene expression downstream through mostly promoter and enhancer regions. Altered gene expression subsequently enhances the vulnerability of DAergic neurons and eventually activates neuronal apoptosis.

Overall, our integrated genome-wide analysis of H3K27ac distribution and transcriptomic alteration sheds light on our hypothesis that H3K27ac plays a critical role during environmentally induced PD pathogenesis. It provides a potentially novel paradigm for studying the epigenetic effects of chronic neurotoxicant exposure in neurodegeneration and reveals H3K27ac as a key epigenetic PTM marker in environmentally linked PD. Through a series of biochemical studies, epigenomic profiling, and bioinformatic identification, as summarized in Figure 10, our results systematically reveal how mitochondrial TFAM variants or environmental insults can trigger a cascade of events in the mitochondrial dysfunction-metabolism-H3K27ac/active enhancer activity-transcriptome axis that contribute to PD pathogenesis (Figure 10).

## Methods

### Chemicals and reagents.

RPMI, neurobasal medium, FBS, L-glutamine, IR dye–tagged secondary antibodies, Hoechst nuclear stain, penicillin, streptomycin, horse serum, and other cell culture reagents were purchased from Thermo Fisher Scientific. The Bradford protein assay kit was purchased from MilliporeSigma. The Phalloidin antibody and protease and phosphatase inhibitor cocktail were ordered from Thermo Fisher Scientific, acetylation inhibitor cocktail from Santa Cruz Biotechnology, and propidium iodide (PI) from Molecular Probes.

### AcetylScan proteomic profiling.

AcetylScan proteomic profiling followed the standard protocol as described by Svinkina et al. (54). At least 10 T175 flasks of N27 DAergic neuronal cells (seeding number 6 × 10^6^) of the same cell passage were grown for each sample (*n* = 2) with 2 × 10^8^ of N27 cells being cultured as one sample. After growing to 70% confluence, cell viability and subconfluence were inspected by microscopy. Next, cells were treated with 1 μM rotenone for 3 hours. After treatment, cell harvesting proceeded until 10 flasks of cells had been collected. Adherent cell cultures were washed with 10 mL of 4°C PBS. After pipetting off all PBS, 10 mL urea lysis buffer (ULB; 20 mM HEPES, pH 8.0, 9.0 M Pierce Sequanal grade urea [29700, Cell Signaling Technology], 1 mM sodium orthovanadate [activated], 2.5 mM sodium pyrophosphate, and 1 mM β-glycerol-phosphate) was added to flask 1 before scraping off the cells. For the remaining 9 flasks, steps were the same except the ULB came from the previous flask (e.g., flask 2 used ULB containing proteins from flask 1). Protein was digested and desalted using preconditioned C18 solid-phase extraction. Peptides were then eluted from antibody beads with 50 μL 0.15% trifluoroacetic acid and completely dried by lyophilization. Next, peptides were resuspended and enriched by motif anti-Kac antibodies noncovalently coupled to protein A–agarose beads and triple-washed with 1.5 mL buffer (13416, Cell Signaling Technology). After immunoprecipitation of beads, the resin was washed and peptides were eluted from it. Peptide fractions were analyzed by online nanoflow LC-MS/MS. Technical triplicates were run per sample. MS parameter settings were as follows: MS run time 96 minutes, MS1 scan range 300.0–1500.00, Top 20 MS/MS (min signal 500, isolation width 2.0, normalized collision energy 35.0, activation-Q 0.250, activation time 20.0, lock mass 371.101237, charge state rejection enabled, charge state 1+ rejected, dynamic exclusion enabled, repeat count 1, repeat duration 35.0, exclusion list size 500, exclusion duration 40.0, exclusion mass width relative to mass, and exclusion mass width 10 ppm). For data analysis, sequences were assigned to MS/MS spectra with Sorcerer for relative quantitation. MS/MS spectra were evaluated using SEQUEST and the core platform from CST. Searches were performed against the most recent update of the NCBI database with a mass accuracy of ±50 ppm for precursor ions and ±1 Da for productions. Results were filtered with a mass accuracy of ±5 ppm for precursor ions and the presence of the intended motif. Data are presented as fold change in protein types using a 2.5-fold cutoff as the minimum effect size.

### Animal studies.

All mice used for this study were bred and maintained under a 12-hour light/12-hour dark cycle in a climate-controlled mouse facility (22°C ± 1°C) with food and water available ad libitum. MP Tg mice were a gift from Nils-Goran Larsson at the Karolinska Institute in Stockholm (87, 88), and were originally generated in his laboratory at the Max Planck Institute for Biology of Ageing by conditionally knocking out TFAM through control of the dopamine transporter (DAT), as previously described. All MP mice used for this study were bred from the MP breeding colony at Iowa State University (ISU) and genotyped to confirm their identity. Equal numbers of male and female animals were allocated to experiment groups comprising 16- to 20-week age-matched C57BL/6 mice (TFAM*^+/LoxP^*: Dat^+/+^ from MP litters or parental-strain litters) or MP mice (Dat^+/Cre^: TFAM*^LoxP/LoxP^*), which were further characterized using VersaMax for monitoring locomotor activity and RotaRod for testing coordination of movement. Regarding VersaMax, mice were monitored for 10 minutes during the daytime phase of the light cycle. For RotaRod, speed was set to 20 rpm and time spent on the rod was measured for 10 minutes maximum during a total of 3 trials.

### Postmortem human PD brain samples.

Freshly frozen SN tissue blocks and cryostat sections from the brains of confirmed postmortem human PD patients and age-matched neurologically normal individuals were obtained from the brain banks at the Miller School of Medicine, University of Miami, Florida, and the Banner Sun Health Research Institute, Arizona. All postmortem human samples were procured, stored, and distributed according to the applicable regulations and guidelines involving consent, protection of human subjects, and donor anonymity as described previously (89). For immunoblotting experiments, tissue homogenates from freshly frozen tissue blocks were prepared at a final concentration of 1 mg/mL total protein from which 35 μg total protein was directly used; or total histone was extracted from tissue blocks and 15–45 μg of total histone was used. For IHC experiments, cryostat sections were stained as described below, but with slight modification. Antigen retrieval was done in citraconic anhydride buffer (0.05%, pH 7.4) at 80°C.

### Gene expression validation by RT-qPCR.

Total RNAs were isolated and reverse transcribed into cDNAs by using a PrimeScript RT reagent kit with gDNA Eraser (Takara Bio Inc.). Five genes related to mitochondrial and neurological functions were selected for RT-qPCR; primers are listed in Supplemental Table 3. All qPCR reactions were performed on an Applied Biosystems StepOne Real-time PCR system (Thermo Fisher Scientific) using iTaq Universal SYBR Green Supermix (Bio-Rad), with 3 technical replicates. The amplification procedure was as follows: 95°C for 5 minutes, followed by 40 cycles at 95°C for 10 seconds and 60°C for 20 seconds. Relative quantification of target genes was performed using the ΔΔC_t_ method with GAPDH as a reference gene.

### RNA-Seq library construction and sequencing.

Total RNAs from treatment and control groups were isolated and quality controlled according to the Illumina protocols. A total of 2 μg RNA per sample was used as initial material for library construction. Poly-A–containing mRNA molecules were purified by using poly-T oligo-attached magnetic beads and fragmented into small pieces using divalent cations under elevated temperature. The cleaved RNA fragments are copied into the first-strand cDNA using reverse transcriptase and random primers. Strand specificity is achieved by replacing dTTP with dUTP, followed by second-strand cDNA synthesis. These cDNA fragments then have the addition of a single adenine base and subsequent ligation of the adapter. The products are then purified and enriched with PCR to create the final cDNA library. RNA-Seq was performed on an Illumina HiSeq ×10 platform and 150-bp single-end reads were generated according to Illumina’s protocol. RNA-Seq data were submitted to NCBI’s Gene Expression Omnibus database (GEO GSE140524).

### Whole-genome gene expression analysis.

Data analysis followed the procedure of Li et al. (90). The adapter sequences were removed from the raw sequencing data, and the individual libraries were converted to the FASTQ format. Sequence reads were aligned to the rat genome (rn6) with HISAT2 (v2.1.0), and the resulting alignment files were reconstructed with the EdgeR3 package. The Ref-Seq database (build 37.3) was chosen as the annotation reference for mRNA analysis. The read counts of each transcript were normalized to the length of the individual transcript, and the total mapped fragments were counted in each sample and expressed as reads per kilobase of exon per million fragments mapped of mRNAs in each sample. The mRNA differential expression analysis was applied to treatment and control groups. An adjusted *P* value of less than or equal to 0.05 (Student’s *t* test with Benjamini-Hochberg FDR adjustment) was used as the cutoff for statistically significantly DEGs. DEGs were analyzed by enrichment analysis to detect overrepresented functional terms present in the genomic background. GO and KEGG pathway analyses were performed using DAVID Bioinformatics Resources 6.8 (18, 91, 92).

### ChIP-Seq.

ChIP was performed according to Wang et al. (26). Briefly, cells were cross-linked with 1% formaldehyde for 10 minutes at room temperature and quenched with 125 mM glycine for 10 minutes. After fragmenting into 100–1000 bp by sonication, chromatin templates from 3 million cells were used for ChIP assay. Samples were immunoprecipitated with 2 μg H3K27ac antibody overnight at 4°C followed by washing steps. After reverse cross-linking, ChIP-DNA fragments were purified and constructed into libraries by using the ThruPLEX DNA-Seq Kit (Rubicon Genomics). Amplified libraries around 300–500 bp were isolated from agarose gels before sequencing. Sequencing was performed on an Illumina HiSeq ×10 platform and 150-bp single-end reads were generated according to Illumina’s protocol. ChIP-Seq data were submitted to the GEO database (GSE140524).

### Identification of H3K27ac regions and peaks.

ChIP-Seq reads were mapped to the rat genome (rn6) using Bowtie aligner, allowing up to 2 mismatches. Only the uniquely mapped reads were used for further analysis. The SICER call peak program was used to call the peaks with a window size of 200 bp and a gap size of 400 bp, and an E value of 1. The EdgeR3 program was used to identify the differential peaks between treatment and control groups (cutoff: FC > 2 and FDR < 0.01).

### ChIP-qPCR assay.

Four gene regions relevant to mitochondrial and neurological function were selected for ChIP-qPCR assay performed using primers listed in Supplemental Table 3. All qPCR reactions were performed on an Applied Biosystems StepOne Real-time PCR system (Thermo Fisher Scientific) using iTaq Universal SYBR Green Supermix (Bio-Rad) with 2 technical replicates. The amplification procedure was as follows: 95°C for 5 minutes, followed by 40 cycles at 95°C for 10 seconds and 60°C for 20 seconds. Relative quantification of target gene regions was performed by normalizing the threshold cycle (C_t_) of samples to the percentage of input DNA.

### Enrichment analysis of transcription factor motifs.

The HOMER ChIP-Seq pipeline was applied for analyzing motif enrichment (93). Motif models were established from the TRANSFAC vertebrate database (94), and the enrichment analysis was performed on rotenone-upregulated and TFAM-KO–upregulated enhancer peaks with all peaks as the background. Enriched motifs were classified into known motifs and de novo motifs.

### SYBR Green RT-qPCR.

RNA was isolated from slices following the manufacturer’s protocol (Thermo Fisher Scientific). In brief, slices were lysed in 1 mL TRIzol reagent and incubated for 5 minutes at room temperature. Next, 0.2 mL chloroform was added for an additional 2-minute incubation. Following centrifugation, the top layer containing RNA was transferred to a new tube containing 0.7 mL isopropanol. After a 15-minute incubation and centrifugation again, RNA pellets were washed with 75% ethanol, air dried, and dissolved in water. The high-capacity cDNA synthesis kit from Applied Biosystems (4368814) was used for cDNA synthesis according to the manufacturer’s protocol. For normalization of each sample in RT-qPCR, the *18S* rRNA gene (PPM57735E, Qiagen) was used as the housekeeping gene. Based on the manufacturer’s guidelines, dissociation and melting curves were run to ensure that single amplicon peaks were obtained without any nonspecific amplicons. Fold change in gene expression was determined using the ΔΔC_t_ method. Quantitative PCR was performed using RT^2^-qPCR SYBR Green Mastermix (Agilent).

### Study approval.

All animal studies followed animal procedures approved by ISU’s IACUC (Ames, IA, USA). For human samples, since the postmortem human brain tissues were obtained from approved national brain banks, IRB approval from ISU was not required.

### Statistics.

Data analysis was performed using GraphPad Prism 7.0. Normally distributed raw data were analyzed with either Student’s *t* test (2-group comparisons) or 1-way ANOVA (>2-group comparisons) with Tukey’s post hoc test unless otherwise mentioned. A *P* value of less than or equal to 0.05 was considered significant: *P ≤ 0.05, **P < 0.01, ***P < 0.001, ****P < 0.0001.

## Author contributions

AK, ZW, and AGK jointly conceived this project and supervised the experiments. MH, AK, ZW, and AGK designed the research. MH and DL performed experiments. MH and HJ analyzed the experimental results. DK and DL accomplished the bioinformatic analyses. MH, DL, AC, DK, HJ, GZ, VA, AK, ZW, and AGK prepared the manuscript. ZW and AGK made the final call (data interpretation and presentation) on epigenomic and animal analyses, respectively.

## Supplementary Material

Supplemental data

Supplemental Table 1

Supplemental Table 2

## Figures and Tables

**Table 1:**
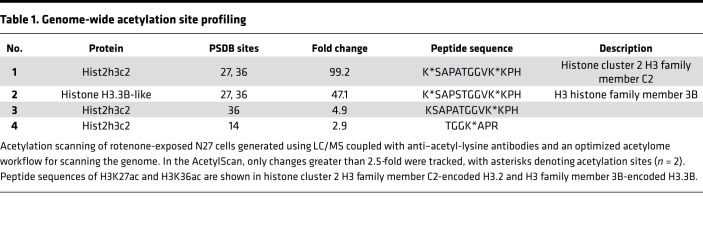
Genome-wide acetylation site profiling

## Version 1 09/08/2021

Electronic publication

## References

[1] Polymeropoulos MH (1997). Mutation in the alpha-synuclein gene identified in families with Parkinson’s disease. Science.

[2] Goldman SM (2014). Environmental toxins and Parkinson’s disease. Annu Rev Pharmacol Toxicol.

[3] Dawson TM (2010). Genetic animal models of Parkinson’s disease. Neuron.

[4] Trancikova A (2012). Mitochondrial dysfunction in genetic animal models of Parkinson’s disease. Antioxid Redox Signal.

[5] Zanon A (2018). Environmental and genetic variables influencing mitochondrial health and Parkinson’s disease penetrance. Parkinsons Dis.

[6] Wu S (2018). Mutation of hop-1 and pink-1 attenuates vulnerability of neurotoxicity in C. elegans: the role of mitochondria-associated membrane proteins in Parkinsonism. Exp Neurol.

[7] Li N (2003). Mitochondrial complex I inhibitor rotenone induces apoptosis through enhancing mitochondrial reactive oxygen species production. J Biol Chem.

[8] Cacabelos R (2017). Parkinson’s disease: from pathogenesis to pharmacogenomics. Int J Mol Sci.

[9] Song C (2010). Environmental neurotoxic pesticide increases histone acetylation to promote apoptosis in dopaminergic neuronal cells: relevance to epigenetic mechanisms of neurodegeneration. Mol Pharmacol.

[10] Song C (2011). Paraquat induces epigenetic changes by promoting histone acetylation in cell culture models of dopaminergic degeneration. Neurotoxicology.

[11] Harrison IF (2018). Pathological histone acetylation in Parkinson’s disease: neuroprotection and inhibition of microglial activation through SIRT 2 inhibition. Neurosci Lett.

[12] Song C (2019). Mechanistic interplay between autophagy and apoptotic signaling in endosulfan-induced dopaminergic neurotoxicity: relevance to the adverse outcome pathway in pesticide neurotoxicity. Toxicol Sci.

[13] Wang Z (2008). Combinatorial patterns of histone acetylations and methylations in the human genome. Nat Genet.

[14] Heintzman ND (2009). Histone modifications at human enhancers reflect global cell-type-specific gene expression. Nature.

[15] Marzi SJ (2018). A histone acetylome-wide association study of Alzheimer’s disease identifies disease-associated H3K27ac differences in the entorhinal cortex. Nat Neurosci.

[16] Borrageiro G (2018). A review of genome-wide transcriptomics studies in Parkinson’s disease. Eur J Neurosci.

[17] Li YI (2019). Prioritizing Parkinson’s disease genes using population-scale transcriptomic data. Nat Commun.

[18] Valentine MNZ (2019). Multi-year whole-blood transcriptome data for the study of onset and progression of Parkinson’s disease. Sci Data.

[19] Matilainen O (2017). Mitochondria and epigenetics — crosstalk in homeostasis and stress. Trends Cell Biol.

[20] Minocherhomji S (2012). Mitochondrial regulation of epigenetics and its role in human diseases. Epigenetics.

[21] Jin H (2014). Histone hyperacetylation up-regulates protein kinase Cδ in dopaminergic neurons to induce cell death: relevance to epigenetic mechanisms of neurodegeneration in Parkinson disease. J Biol Chem.

[22] Jin H (2011). α-Synuclein negatively regulates protein kinase Cδ expression to suppress apoptosis in dopaminergic neurons by reducing p300 histone acetyltransferase activity. J Neurosci.

[23] Miyazaki T (2012). Intracellular and extracellular ATP coordinately regulate the inverse correlation between osteoclast survival and bone resorption. J Biol Chem.

[24] Langley MR (2018). Manganese exposure exacerbates progressive motor deficits and neurodegeneration in the MitoPark mouse model of Parkinson’s disease: relevance to gene and environment interactions in metal neurotoxicity. Neurotoxicology.

[25] Curry DW (2018). Targeting AMPK signaling as a neuroprotective strategy in Parkinson’s disease. J Parkinsons Dis.

[26] Wang Z (2009). Genome-wide mapping of HATs and HDACs reveals distinct functions in active and inactive genes. Cell.

[27] Hin N (2020). Accelerated brain aging towards transcriptional inversion in a zebrafish model of the K115fs mutation of human PSEN2. PLoS One.

[28] van der Werf IM (2017). Mutations in two large pedigrees highlight the role of ZNF711 in X-linked intellectual disability. Gene.

[29] Kopp A (2012). Dmrt genes in the development and evolution of sexual dimorphism. Trends Genet.

[30] Grillner S, El Manira A (2020). Current principles of motor control, with special reference to vertebrate locomotion. Physiol Rev.

[31] Andersson LS (2012). Mutations in DMRT3 affect locomotion in horses and spinal circuit function in mice. Nature.

[32] Hoare S (1999). Identification of a GABP alpha/beta binding site involved in the induction of oxytocin receptor gene expression in human breast cells, potentiation by c-Fos/c-Jun. Endocrinology.

[33] Manna PR (2002). Regulation of steroidogenesis and the steroidogenic acute regulatory protein by a member of the cAMP response-element binding protein family. Mol Endocrinol.

[34] Vlahopoulos SA (2008). The role of ATF-2 in oncogenesis. Bioessays.

[35] Zhang X (2007). HOXC6 and HOXC11 increase transcription of S100beta gene in BrdU-induced in vitro differentiation of GOTO neuroblastoma cells into Schwannian cells. J Cell Mol Med.

[36] Borggrefe T, Oswald F (2009). The Notch signaling pathway: transcriptional regulation at Notch target genes. Cell Mol Life Sci.

[37] Kubota K (2015). Dynamic regulation of AP-1 transcriptional complexes directs trophoblast differentiation. Mol Cell Biol.

[38] Cai L (2011). Acetyl-CoA induces cell growth and proliferation by promoting the acetylation of histones at growth genes. Mol Cell.

[39] Imai S (2000). Transcriptional silencing and longevity protein Sir2 is an NAD-dependent histone deacetylase. Nature.

[40] Perillo B (2008). DNA oxidation as triggered by H3K9me2 demethylation drives estrogen-induced gene expression. Science.

[41] Hino S (2012). FAD-dependent lysine-specific demethylase-1 regulates cellular energy expenditure. Nat Commun.

[42] Sciacovelli M (2016). Fumarate is an epigenetic modifier that elicits epithelial-to-mesenchymal transition. Nature.

[43] Azhibekov TA (2011). TM4SF10 and ADAP interaction in podocytes: role in Fyn activity and nephrin phosphorylation. Am J Physiol Cell Physiol.

[44] Saminathan H (2011). Environmental neurotoxic pesticide dieldrin activates a non receptor tyrosine kinase to promote PKCδ-mediated dopaminergic apoptosis in a dopaminergic neuronal cell model. Neurotoxicology.

[45] Kaul S (2005). Tyrosine phosphorylation regulates the proteolytic activation of protein kinase Cdelta in dopaminergic neuronal cells. J Biol Chem.

[46] Panicker N (2019). Fyn kinase regulates misfolded α-synuclein uptake and NLRP3 inflammasome activation in microglia. J Exp Med.

[47] Ficazzola MA (2001). Antiproliferative B cell translocation gene 2 protein is down-regulated post-transcriptionally as an early event in prostate carcinogenesis. Carcinogenesis.

[48] Lau D, Bading H (2009). Synaptic activity-mediated suppression of p53 and induction of nuclear calcium-regulated neuroprotective genes promote survival through inhibition of mitochondrial permeability transition. J Neurosci.

[49] Berson A (2018). Epigenetic regulation in neurodegenerative diseases. Trends Neurosci.

[50] Labbé C (2016). Epigenetic regulation in Parkinson’s disease. Acta Neuropathol.

[51] Weinert BT (2014). Acetylation dynamics and stoichiometry in Saccharomyces cerevisiae. Mol Syst Biol.

[52] Baeza J (2014). Stoichiometry of site-specific lysine acetylation in an entire proteome. J Biol Chem.

[53] Choudhary C (2014). The growing landscape of lysine acetylation links metabolism and cell signalling. Nat Rev Mol Cell Biol.

[54] Svinkina T (2015). Deep, quantitative coverage of the lysine acetylome using novel anti-acetyl-lysine antibodies and an optimized proteomic workflow. Mol Cell Proteomics.

[55] Gonzalez-Reyes LE (2012). Sonic hedgehog maintains cellular and neurochemical homeostasis in the adult nigrostriatal circuit. Neuron.

[56] Siani F (2017). Influence of estrogen modulation on glia activation in a murine model of Parkinson’s disease. Front Neurosci.

[57] Stephano F (2018). Impaired Wnt signaling in dopamine containing neurons is associated with pathogenesis in a rotenone triggered Drosophila Parkinson’s disease model. Sci Rep.

[58] Kim EK, Choi EJ (2010). Pathological roles of MAPK signaling pathways in human diseases. Biochim Biophys Acta.

[59] Huang M (2019). Paraquat modulates microglia M1/M2 polarization via activation of TLR4-mediated NF-κB signaling pathway. Chem Biol Interact.

[60] Rousseau A, Bertolotti A (2018). Regulation of proteasome assembly and activity in health and disease. Nat Rev Mol Cell Biol.

[61] Bentea E (2017). The proteasome inhibition model of Parkinson’s disease. J Parkinsons Dis.

[62] Iwabu M (2010). Adiponectin and AdipoR1 regulate PGC-1alpha and mitochondria by Ca(2+) and AMPK/SIRT1. Nature.

[63] Koentges C (2015). Myocardial mitochondrial dysfunction in mice lacking adiponectin receptor 1. Basic Res Cardiol.

[64] Sodersten E (2014). Dopamine signaling leads to loss of Polycomb repression and aberrant gene activation in experimental parkinsonism. PLoS Genet.

[65] Xia P (2014). RNF2 is recruited by WASH to ubiquitinate AMBRA1 leading to downregulation of autophagy. Cell Res.

[66] Kim JM (2006). Identification of genes related to Parkinson’s disease using expressed sequence tags. DNA Res.

[67] Plagge A (2008). Physiological functions of the imprinted Gnas locus and its protein variants Galpha(s) and XLalpha(s) in human and mouse. J Endocrinol.

[68] Martos SN (2017). Two approaches reveal a new paradigm of ‘switchable or genetics-influenced allele-specific DNA methylation’ with potential in human disease. Cell Discov.

[69] Aruga J (2003). Human SLITRK family genes: genomic organization and expression profiling in normal brain and brain tumor tissue. Gene.

[70] Utami KH (2014). Detection of chromosomal breakpoints in patients with developmental delay and speech disorders. PLoS One.

[71] Gillies GE (2014). Sex differences in Parkinson’s disease. Front Neuroendocrinol.

[72] Panicker N (2019). Fyn amplifies NLRP3 inflammasome signaling in Parkinson’s disease. Aging (Albany NY).

[73] Saminathan H (2020). Fyn kinase mediates pro-inflammatory response in a mouse model of endotoxemia: relevance to translational research. Eur J Pharmacol.

[74] Creyghton MP (2010). Histone H3K27ac separates active from poised enhancers and predicts developmental state. Proc Natl Acad Sci U S A.

[75] Fu S (2018). Differential analysis of chromatin accessibility and histone modifications for predicting mouse developmental enhancers. Nucleic Acids Res.

[76] Kumar V (2013). Uniform, optimal signal processing of mapped deep-sequencing data. Nat Biotechnol.

[77] Ernst J (2011). Mapping and analysis of chromatin state dynamics in nine human cell types. Nature.

[78] Maurano MT (2012). Systematic localization of common disease-associated variation in regulatory DNA. Science.

[79] Yuniati L (2019). Tumor suppressors BTG1 and BTG2: beyond growth control. J Cell Physiol.

[80] Gómez-Pinilla F, Cotman CW (1993). Distribution of fibroblast growth factor 5 mRNA in the rat brain: an in situ hybridization study. Brain Res.

[81] Lindholm D (1994). Fibroblast growth factor-5 promotes differentiation of cultured rat septal cholinergic and raphe serotonergic neurons: comparison with the effects of neurotrophins. Eur J Neurosci.

[82] Tiwari PC, Pal R (2017). The potential role of neuroinflammation and transcription factors in Parkinson disease. Dialogues Clin Neurosci.

[83] Sun W (2016). Histone acetylome-wide association study of autism spectrum disorder. Cell.

[84] Niu W (2018). Phenotypic reprogramming of striatal neurons into dopaminergic neuron-like cells in the adult mouse brain. Stem Cell Reports.

[85] Lim DA, Alvarez-Buylla A (2016). The adult ventricular-subventricular zone (V-SVZ) and olfactory bulb (OB) neurogenesis. Cold Spring Harb Perspect Biol.

[86] Toker L, et al. Dysregulation of histone acetylation and decoupling from transcription in Parkinson’s disease [preprint]. 10.1101/785550 Posted on bioRxiv September 28, 2019

[87] Ekstrand MI (2007). Progressive parkinsonism in mice with respiratory-chain-deficient dopamine neurons. Proc Natl Acad Sci U S A.

[88] Ekstrand MI, Galter D (2009). The MitoPark mouse — an animal model of Parkinson’s disease with impaired respiratory chain function in dopamine neurons. Parkinsonism Relat Disord.

[89] Gordon R (2016). Prokineticin-2 upregulation during neuronal injury mediates a compensatory protective response against dopaminergic neuronal degeneration. Nat Commun.

[90] Li Z (2015). Distinct roles of DNMT1-dependent and DNMT1-independent methylation patterns in the genome of mouse embryonic stem cells. Genome Biol.

[91] Huang DW (2009). Bioinformatics enrichment tools: paths toward the comprehensive functional analysis of large gene lists. Nucleic Acids Res.

[92] Kanehisa M, Goto S (2000). KEGG: kyoto encyclopedia of genes and genomes. Nucleic Acids Res.

[93] Heinz S (2010). Simple combinations of lineage-determining transcription factors prime cis-regulatory elements required for macrophage and B cell identities. Mol Cell.

[94] Matys V (2006). TRANSFAC and its module TRANSCompel: transcriptional gene regulation in eukaryotes. Nucleic Acids Res.

