# Supplementary material for: Mitochondrial dysfunction–induced H3K27 hyperacetylation perturbs enhancers in Parkinson’s disease
Source: JCI Insight. 2021 Sep 8;6(17):e138088. doi: 10.1172/jci.insight.138088 (PMC8492320; doi:10.1172/jci.insight.138088)
Supplement: Supplemental data [file jciinsight-6-138088-s185.pdf]

Supplementary Materials

Mitochondrial Dysfunction-Induced H3K27 Hyperacetylation Perturbs Enhancers in Parkinson's Disease

Authors

Minhong Huang[1 †], Dan Lou[2 †], Adhithiya Charli[1], Dehui Kong[2,3], Huajun Jin[1], Gary Zenitsky[1], Vellareddy Anantharam[1], Arthi Kanthasamy[1], Zhibin Wang[2,3*], Anumantha G. Kanthasamy[1*]

Affiliations

[1]Parkinson Disorders Research Laboratory, Iowa Center for Advanced Neurotoxicology, Department of Biomedical Sciences, 2062 Veterinary Medicine Building, Iowa State University, Ames, IA 50011

[2]Laboratory of Environmental Epigenomes, Department of Environmental Health and Engineering, Bloomberg School of Public Health, Johns Hopkins University, Baltimore, MD 21205

[3]State Key Laboratory of Biocatalysis and Enzyme Engineering, College of Life Sciences, Hubei University, Wuhan, Hubei Province 430062, China

[†]These authors contributed equally to this work.

*To whom correspondence should be addressed: Anumantha Kanthasamy, Ph.D., Distinguished Professor and Lloyd Endowed Chair, Department of Biomedical Sciences, Iowa State University, Ames, IA 50011. Telephone: (515) 294-2516; Fax: (515) 294-2315; Email: akanthas@iastate.edu

Zhibin Wang, Ph.D., Associate Professor, Laboratory of Environmental Epigenomes, Department of Environmental Health and Engineering, Bloomberg School of Public Health, Johns Hopkins University, Baltimore, MD 21205. Phone: (410) 955-7840; Email: zwang47@jhu.edu

Supplementary Materials

Materials and Methods

CRISPR/Cas9-based TFAM-KO stable cell generation

The lentivirus-based CRISPR/Cas9 TFAM-KO method follows Gordon *et al*. (78). The TFAM-KO plasmid pLV-U6gRNA-Ef1aPuroCas9GFP-TFAM, with the TFAM-gRNA target sequence directed against the exon 1 sequence (CPR555e5e4099bf84.98), was purchased from Sigma-Aldrich. The lenti-CRISPR/Cas9 TFAM-KO plasmid and universal negative control lentivirus (U6-gRNA/CMV-Cas9-GFP) were respectively transfected into 293FT cells using the Mission Lentiviral Packaging Mix (Cat#SHP001, Sigma-Aldrich) according to manufacturer's instructions. The lentivirus was harvested 48 h after transfection and added to N27 cells for infection at an MOI of 100. After 24 h, puromycin (50 μg/mL) was supplemented for stable cell selection.

Mitochondrial membrane potential, morphology, and superoxide production

The JC-1 mitochondrial potential sensor was purchased from Invitrogen and the standard commercial protocol was followed. Briefly, 2.0 μg/mL of JC-1 diluted in serum-free N27 media was added to N27 cell cultures and incubated at 37°C for 20 min. Following gentle, triple washes with PBS, images were immediately captured on the Keyence microscope (Itasca, IL) before cells dried out. The ratio of red to green was calculated. For MitoTracker staining, procedures were similar. Following the treatment paradigm, 300 μL of 166 nM CMXROS MitoTracker red dye diluted in serum-free N27 media was added and incubated at 37°C for 12 min. After triple-washing with PBS, cells were fixed in a 4% solution of paraformaldehyde (PFA) for 30 min prior to following the steps for ICC and 3D imaging. For MitoSox, live N27 cells were stained following the manufacturer's instructions to detect superoxide production.

Mitochondrial bioenergetics analysis

To measure mitochondrial oxygen consumption and extracellular acidification rates, a Seahorse XFe24 analyzer was used for the Mito Stress test. Following Panicker *et al*. (39), 0.75 μM oligomycin, 1 μM carbonyl cyanide p-trifluoromethoxyphenylhydrazone, and 0.5 μM rotenone-antimycin, serum-free medium were used.

Immunoblotting

Cells were lysed by homogenizing and sonicating in modified RIPA buffer, and then centrifuged. For tissues from toxicant-treated and MP mice, slices and SN blocks were lysed following the same process but without sonication. Proteins were normalized using Bradford assay before loading on sodium dodecyl sulfate (SDS)-acrylamide gels. For separation of proteins, 20-40 μg of protein was loaded in each well of 15% acrylamide gels. The primary antibodies used are as follows: H3K27ac (8173, Cell Signaling, 1:1000), TFAM (sc-166965, Santa Cruz, 1:1000), and H3 (06-755, Millipore, 1:1000), each with β-actin (A2228, Sigma, 1:10000) as loading control. The secondary antibodies used are as follows: IR-800 conjugated goat anti-mouse IgG (LI-COR, 1:20000), IR-700 conjugated goat anti-rabbit IgG (LI-COR 1:20000), and IR-800 conjugated donkey anti-goat IgG (LI-COR, 1:20000).

ICC

For ICC, 4% PFA was used to fix N27 DAergic neuronal cells. After a double-wash, fixed cells were blocked and then incubated in primary antibodies following manufacturers' protocols. Following primary antibody (H3K27ac 1:500, Cell Signaling Technology) incubation, cells were washed with PBS and incubated in Alexa dye-conjugated secondary antibody. Next, cells were washed and mounted on slides using Fluoromount aqueous mounting medium (Sigma). Cells were visualized using an inverted fluorescence microscope (Nikon TE-2000U).

Acute midbrain slice

Organotypic slices were prepared as previously described with several steps improved to adapt to a PD disease model (86). The slice culture buffer comprised Gey's balanced salt solution supplemented with the excitotoxic antagonist kynurenic acid (GBSS). WT (C57BL/6) postnatal mouse pups (7- to 12-days old) were used for live brain slices. The microtome's (Compresstome VF-300, Precisionary Instruments) slicing reservoir was prefilled with ice-cold sterile GBSS. The dissected midbrain was oriented in the sagittal plane in the Compresstome's specimen tube with 2% liquid agarose inside. After quickly solidifying the agar, the specimen tube was inserted into the slicing reservoir. Nigrostriatal slices (200 μm) were transferred to 6-well plates with oxygenated GBSS. The plates were preheated to 37°C in a humidified atmosphere of 5% CO2. After a 1- to 2-h recovery, acute slices were treated with 25 μM rotenone for 3 h. Afterward, slices were gently double-washed in ice-cold PBS, temporarily stained with propidium iodide to ensure viability and homogenized to prepare lysates for Western blotting or RNA for qRT-PCR. Slices were exposed to rotenone treatment in 6-well plates in an incubator maintained at 5% CO2 and 37°C. Treatments were performed in serum-free RPMI media supplemented with penicillin (100 U/mL), streptomycin (100 ug/mL), and 2 mM L-glutamine. After a 3-h treatment, slices were washed with ice-cold PBS and harvested.

Table S1. Rotenone and TFAM-KO DEGs (Please find the Excel file 'Suppl Table S2-Rotenone&TFAM_DEGs' attached).

Table S2. Rotenone and TFAM known motifs (Please find the Excel file 'Suppl Table S3-Rotenone&TFAM_knownMotifs' attached).

Table S3. Primers for qRT-PCR*.

Assay	Primer ID	Sequence (5'-3')
qRT-PCR	Adipor1	F: AGACCACCTATGCCCTCCTT<br>R: TGGAGGGGAGCCATGAAGTA
	Tmem183a	F: GACACAAAGAAGACCTGGATGG<br>R: ACAACCTGGTCCAAAAGGCA
	Gnas	F: TGGACACTGAGAACATCCGC<br>R: TAGAGCAGCTCGTATTGGCG
	Btg2	F: CTGACAACAGGCCACCGTAT<br>R: CTTCCAAGCAGCTCCCGTTA
	Rnf2	F: GCTTGACCAAGTGGTGTGGT<br>R: ACGATACACAGAAGGCCACAG
	GAPDH	F: AGTGCCAGCCTCGTCTCATA<br>R: GATGGTGATGGGTTTCCCGT
ChIP-qPCR	Tmem47	F: CATGGAAGAGGTGCGAGTGT<br>R: TATCCAGGTTGGCGGGTTTC
	Tmem183a	F: GAAACAGAGCAAAGCGCCAA<br>R: GAGAGGAAAGCGGCTCAAGT
	Fgf5	F: CGGCTCGGAACATAGCAGTT<br>R: CGTGGGAGCCATTGACTTTG
	Btg2	F: AACCTTGAGTCTCTGCTCGC<br>R: CACGGGAAGAGAACCGACAT

* Independent qRT-PCR experiments were repeated in two labs for validating gene expression. Primers shown in the table were purchased from Integrated DNA Technologies (Coralville, IA). In the other independent experiment, commerical primers were purchased from QIAGEN (Germantown, MD).

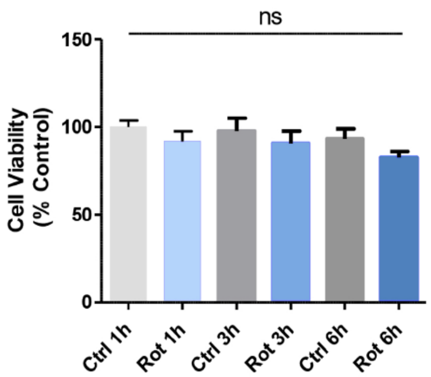

Supplementary Figure 1. MTS assay of cell viability after treating N27 cells with the mitochondrial complex-1 inhibitor rotenone (1 µM) for 1, 3, and 6 h. Data analyzed via one-way ANOVA with Tukey's post hoc test (ns, not significant) and are represented as mean ± SEM with n=8.

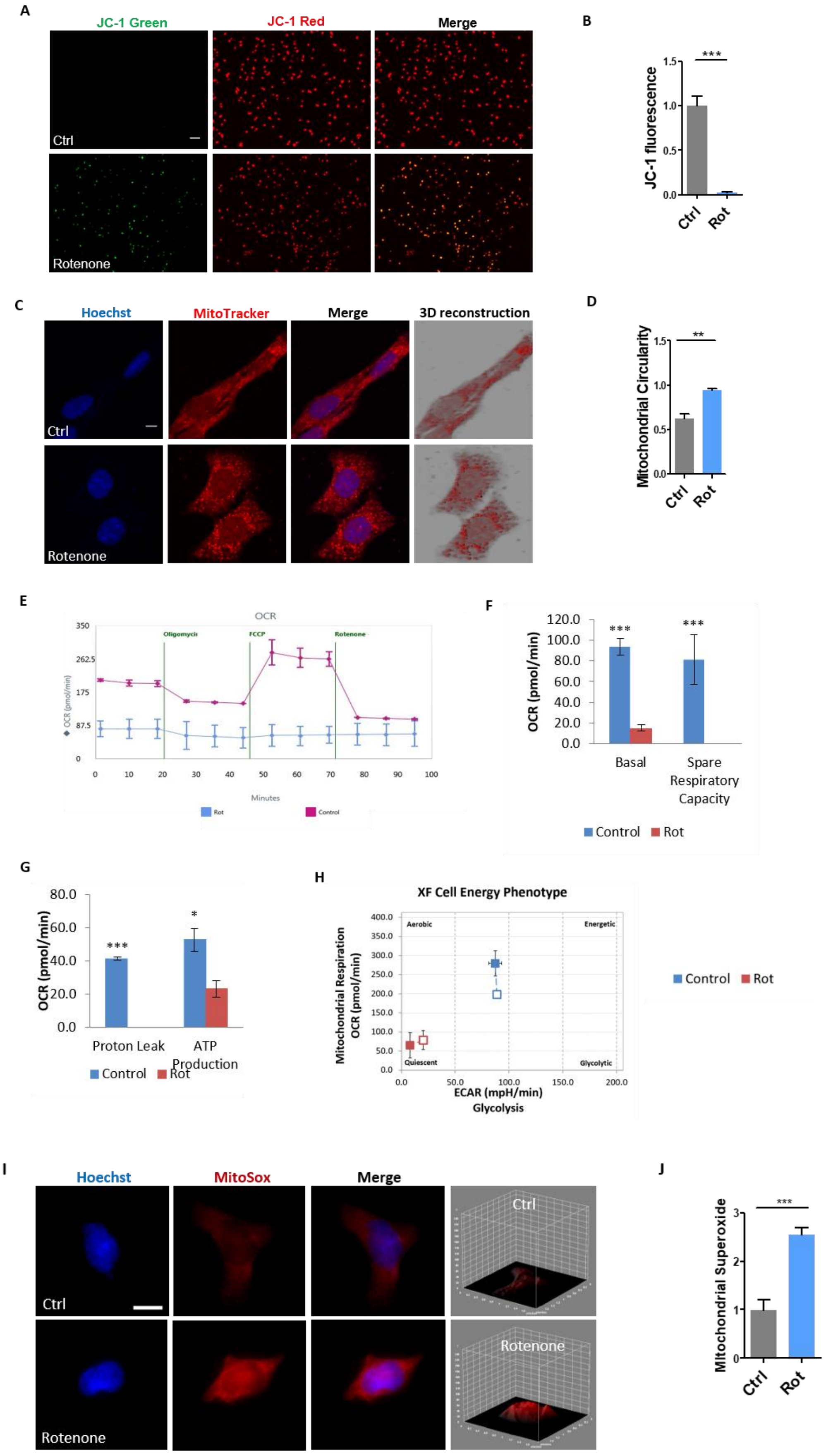

A
JC-1 Green
JC-1 Red
Merge
Ctrl
Rotenone
B
JC-1 fluorescence
***
1.5
1.0
0.5
0.0
Ctrl
Rot
C
Hoechst
MitoTracker
Merge
3D reconstruction
Ctrl
Rotenone
D
Mitochondrial Circularity
**
1.5
1.0
0.5
0.0
Ctrl
Rot
E
OCR
Oligomycin
FCCP
Rotenone
350
262.5
175
87.5
0
0
10
20
30
40
50
60
70
80
90
100
Minutes
Rot
Control
F
OCR (pmol/min)
120.0
100.0
80.0
60.0
40.0
20.0
0.0
***
***
Basal
Spare Respiratory Capacity
Control
Rot
G
OCR (pmol/min)
80.0
60.0
40.0
20.0
0.0
***
*
Proton Leak
ATP Production
Control
Rot
H
XF Cell Energy Phenotype
Mitochondrial Respiration OCR (pmol/min)
400.0
350.0
300.0
250.0
200.0
150.0
100.0
50.0
0.0
Aerobic
Energetic
Quiescent
Glycolytic
0.0
50.0
100.0
150.0
200.0
ECAR (mpH/min)
Glycolysis
Control
Rot
I
Hoechst
MitoSox
Merge
Ctrl
Rotenone
J
Mitochondrial Superoxide
***
3
2
1
0
Ctrl
Rot

Supplementary Figure 2. Rotenone (Rot) exposure increased depolarization of mitochondrial membrane potential in N27 cells. (A) JC-1 probe (scale bar, 100 µm) and (B) quantification of the $\Delta\psi m$ by the ratio of the red and green fluorescence. (C) Mitochondrial morphological changes show up as small, punctate mitochondria, compared to elongated mitochondria in control as measured by MitoTracker (red) dye. Independent experiments were repeated at least three times. Scale bars, 10 µm. (D) The quantification of mitochondrial circularity. (E, F, and G) Impaired mitochondrial bioenergetics via XFe24 Seahorse mitochondrial stress test displayed as (E) Respiration plot, (F) basal respiration and spare respiratory capacity, and (G) proton leak and ATP production. (H) Cellular phenotype plot comparing OCR on the y-axis and ECAR on the x-axis. Three biological replicates for each treatment. (I) Oxidative stress as measured by MitoSox (red) assay showing mitoROS production coupled with 3D plots of MitoSox fluorescence intensity. Independent experiments were repeated at least four times. Scale bars, 10 µm. (J) Quantification of mitochondrial superoxide production. Error bars represent mean±SEM of one-way ANOVA followed by Tukey's post hoc test. Experiments were repeated three times with 3-4 samples per time. In (J), bar graphs show mean ± SEM of unpaired two-tailed t-tests. ns, not significant; *$p \leq 0.05$; **$p < 0.01$; ***$p < 0.001$.

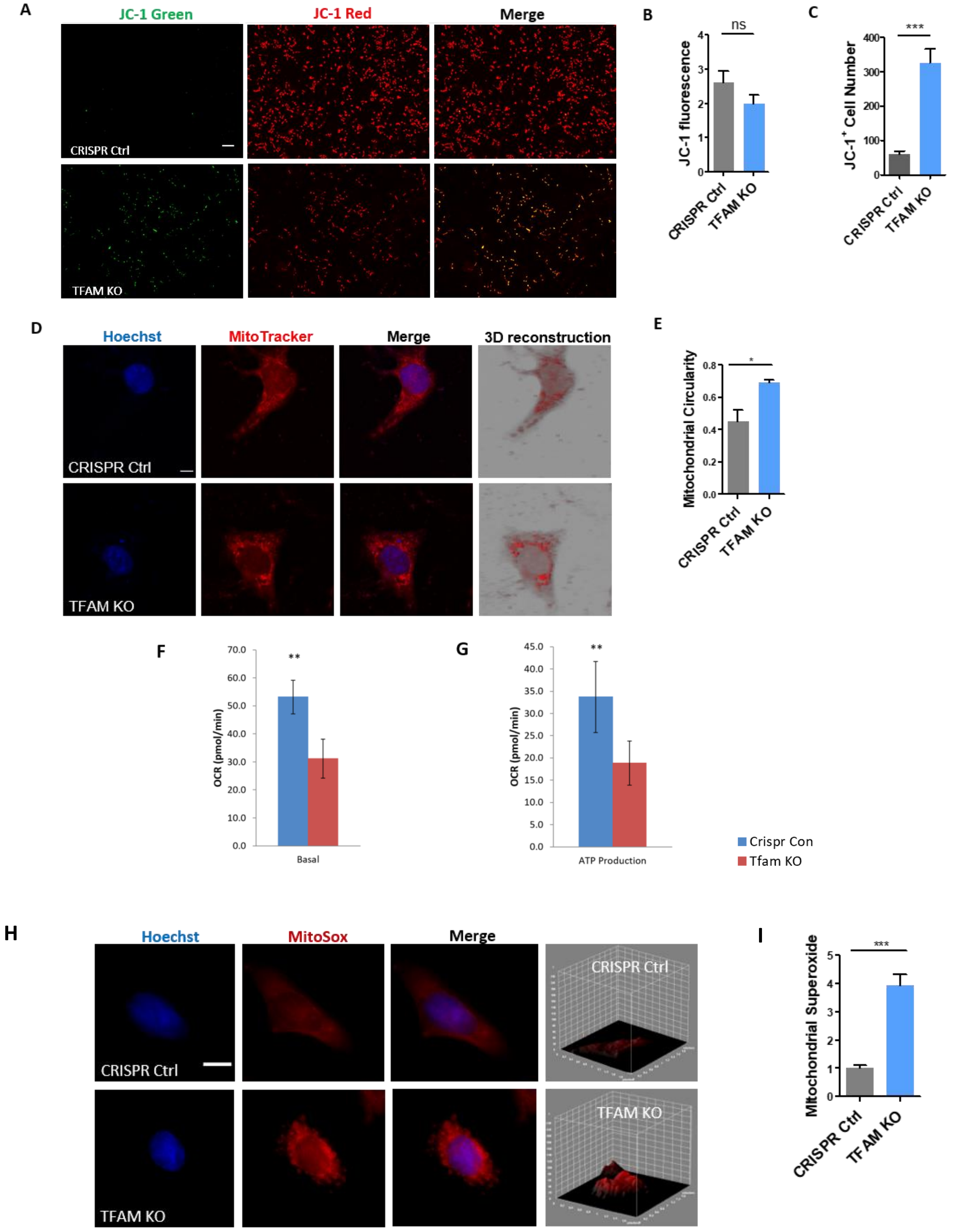
A
JC-1 Green
JC-1 Red
Merge
CRISPR Ctrl
TFAM KO
B
ns
JC-1 fluorescence
CRISPR Ctrl
TFAM KO
C
***
JC-1+ Cell Number
CRISPR Ctrl
TFAM KO
D
Hoechst
MitoTracker
Merge
3D reconstruction
CRISPR Ctrl
TFAM KO
E
*
Mitochondrial Circularity
CRISPR Ctrl
TFAM KO
F
**
OCR (pmol/min)
Basal
G
**
OCR (pmol/min)
ATP Production
Crispr Con
Tfam KO
H
Hoechst
MitoSox
Merge
CRISPR Ctrl
TFAM KO
I
***
Mitochondrial Superoxide
CRISPR Ctrl
TFAM KO

Supplementary Figure 3. TFAM-KO increased depolarization of mitochondrial membrane potential in N27 cells. (A) JC1 probe (scale bar, 100 μm) and (B) quantitative analysis of $\Delta\psi m$ by the ratio of the red and green fluorescence. (C) Cell number of TFAM KO shown in green color. Experiments were repeated three times with 3-4 samples per time. (D) Mitochondrial morphological changes show up as small, punctate mitochondria, compared to elongated mitochondria in control as measured by MitoTracker (red) dye. Independent experiments were repeated at least three times. Scale bars, 10 μm. (E) The quantification of mitochondrial circularity. Bar graph shows mean ± SEM of unpaired two-tailed t-tests with Welch's correction. (F and G) Impaired mitochondrial bioenergetics analyzed by XFe24 Seahorse mitochondrial stress test to show (F) basal respiration and (G) ATP production. Five independent measurements for each data point. Bar graphs show mean ± SEM of unpaired two-tailed t-tests. (H) oxidative stress as measured by MitoSox (red) assay showing mitoROS production coupled with 3D plots of MitoSox fluorescence intensity. Independent experiments were repeated at least four times. Scale bars, 10 μm. (I) Quantification of mitochondrial superoxide production. Error bars represent mean±SEM of one-way ANOVA followed by Tukey's post hoc test. Experiments were repeated three times with 3-4 samples per time. ns, not significant; *$p \leq 0.05$; **$p < 0.01$; ***$p < 0.001$.

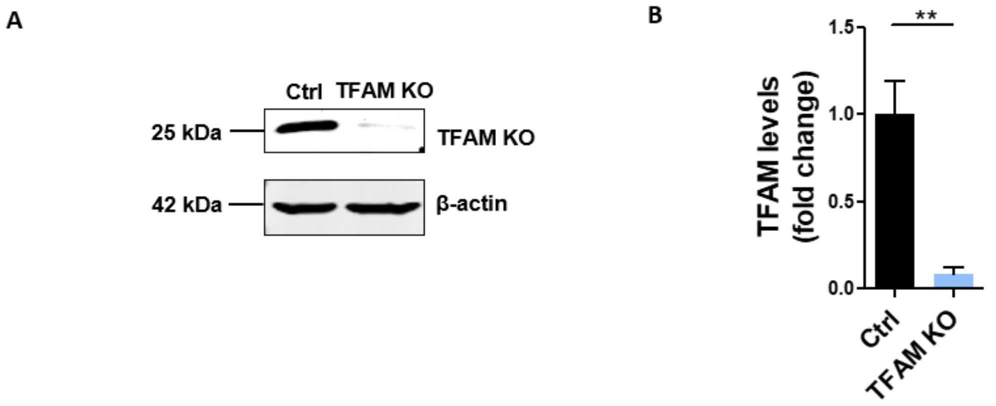

Supplementary Figure 4. Mitochondrial transcription factor A (TFAM) was knocked out in N27 DAergic neuronal cells. (A) Representative immunoblot for TFAM. (B) Densitometric analysis of bands in immunoblot. Bar graph shows mean ± SEM of unpaired two-tailed t-tests with n=4. *p≤0.05, **p<0.01.

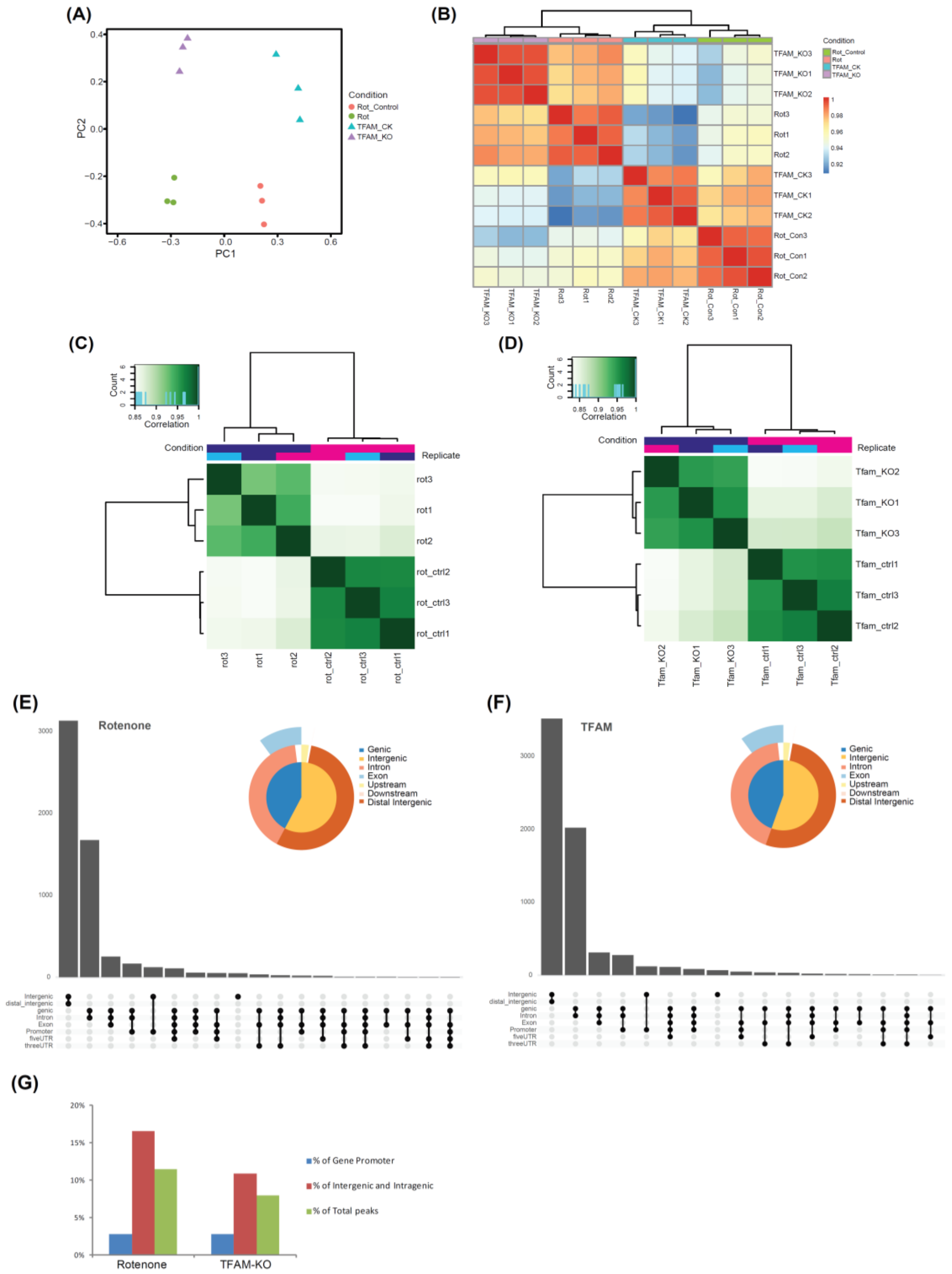

Supplementary Figure 5. (A) Principal component analysis (PCA) of transcriptome profiles in N27 cells with and without rotenone treatment and TFAM knockout. (B-D) Hierarchical clustering of transcriptome profile in N27 cells with and without rotenone treatment and TFAM knockout. (E-F) Upset plots and Venn plots showing the normalized genome-wide H3K27ac distribution in N27 cells with and without (C) rotenone treatment and (D) TFAM knockout. (G) Genome-wide H3K27ac modification alterations in response to mitochondrial impairment induced by (E) rotenone and (F) TFAM KO. Approximately 11.5% of H3K27ac peaks were significantly changed after rotenone treatment with 2.79% and 14.36% of them located at annotated genes (proximal promoters) and distal promoters, respectively. Upon TFAM knockout, 8.0% of H3K27ac peaks were significantly changed with 2.82% and 9.63% of them at annotated genes (proximal promoters) and distal promoters, respectively.

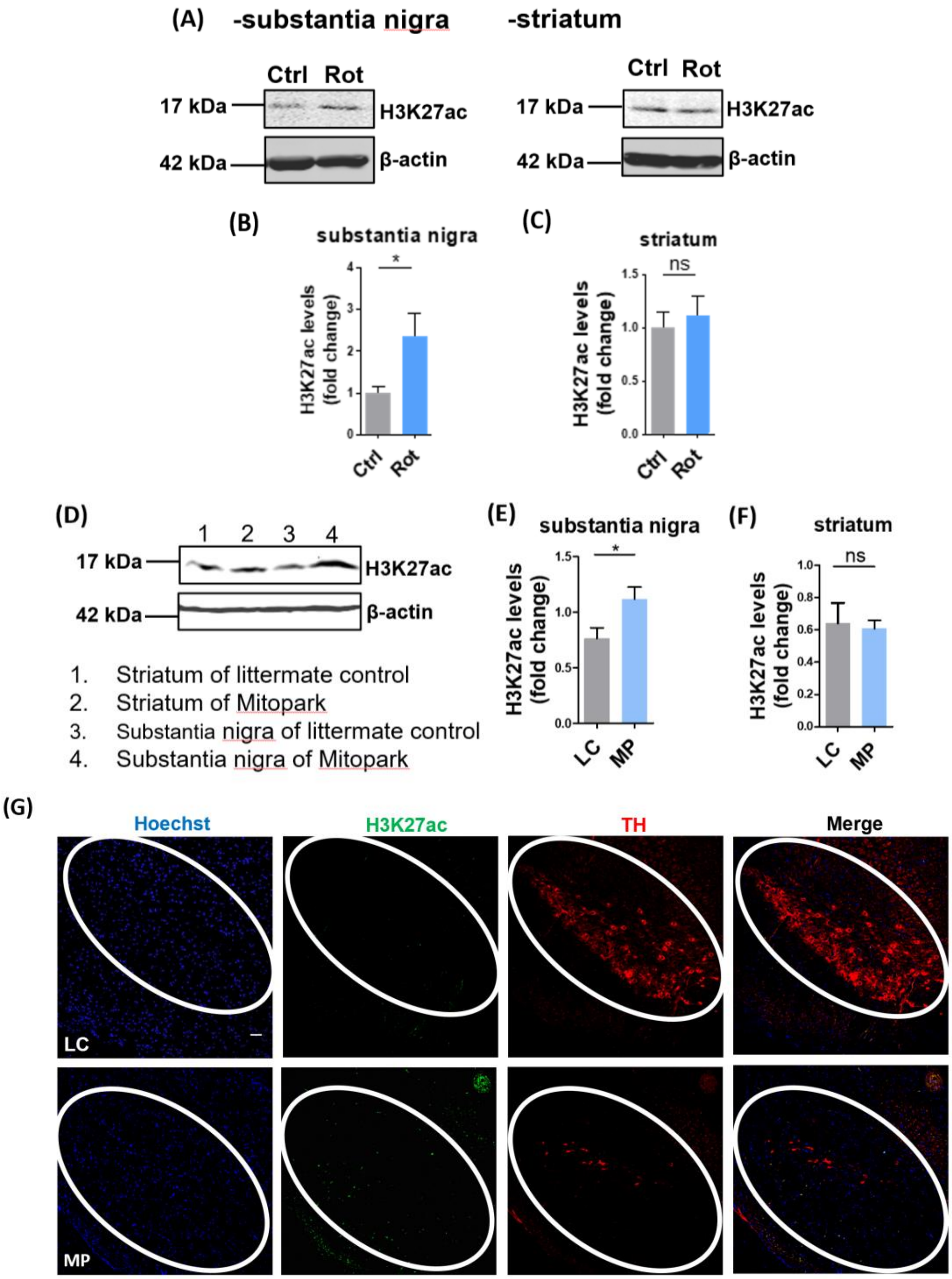

Supplementary Figure 6. Elevated H3K27ac in substantia nigra (SN) of MitoPark mice (MP) and in midbrain slices exposed to rotenone *ex-vivo*. (A) Representative immunoblots for H3K27ac in rotenone-exposed midbrain slices coupled with their densitometric analyses for the (B) SN and (C) striatum. Each data point is the average of three replicates. At least four independent experiments were measured for SN and two for striatum. (D) Representative immunoblot for H3K27ac in the SN (n=11-13) and striatum (n=7) from 16- to 20-week-old littermate controls (LCs) and MPs; β-actin is the internal control. The immunoblot quantification of H3K27ac is shown for (E) the SN and (F) striatum. (G) IHC showing TH degradation for the entire SN region from 16- to 20-week-old LCs and MPs with H3K27ac in green, nucleus stained blue with Hoechst and tyrosine hydroxylase (TH) in red. Scale bar, 50 µm. Independent experiments were repeated four times. Bar graphs show mean ± SEM of unpaired two-tailed t-tests. ns, not significant; *p≤0.05; **p<0.01.

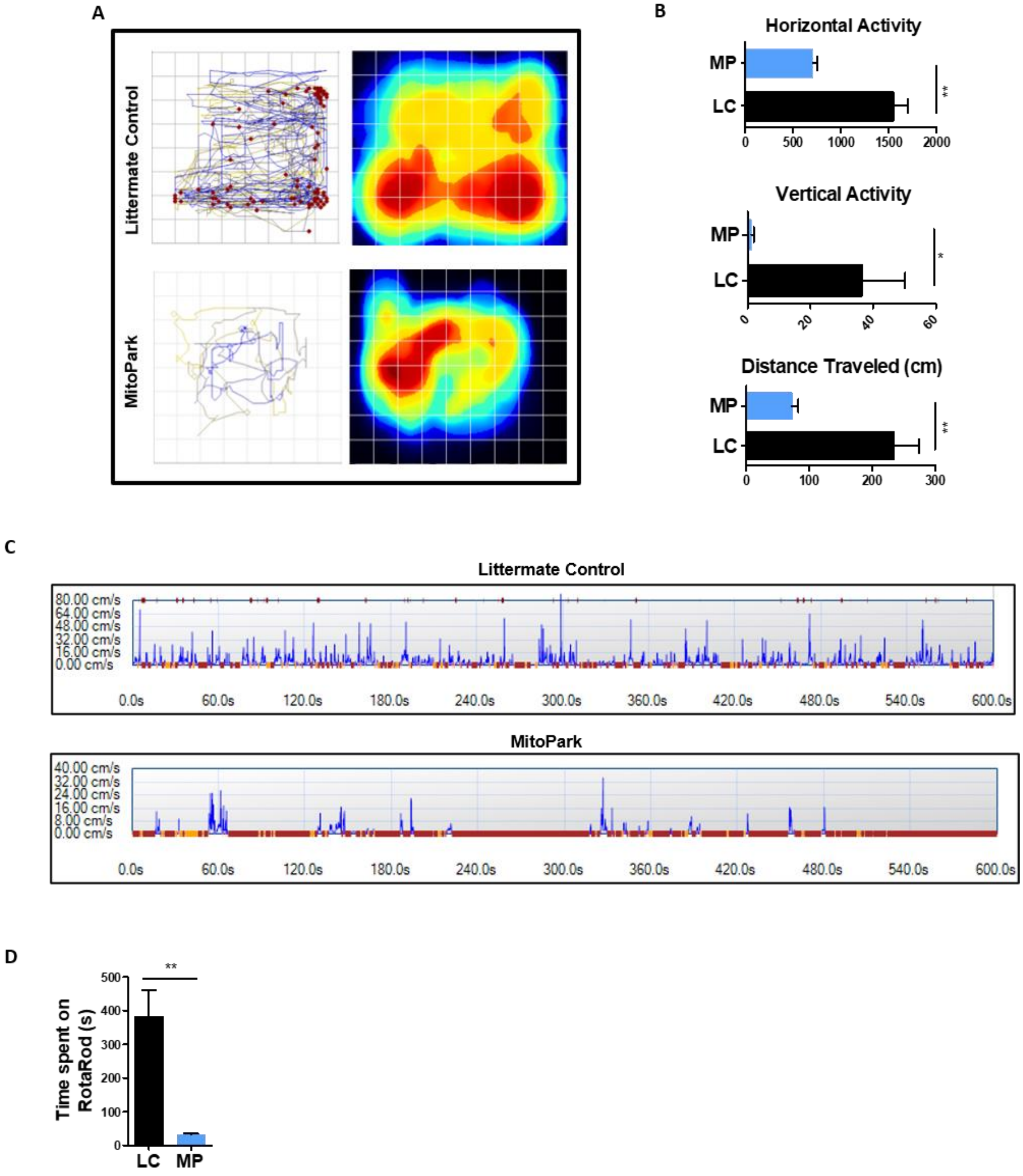

Supplementary Figure 7. All littermate (LC) and MitoPark (MP) mice used in this study were genotyped and further characterized by behavioral phenotyping assays. Motor deficits in MP mice depicted as (A) representative track plots and heat maps generated in VersaPlot, (B) group horizontal activity, vertical activity and distance traveled, (C) representative horizontal movement velocity, and (D) group RotaRod activity. Bar graphs show mean ± SEM of unpaired two-tailed t-tests. ns, not significant; *p≤0.05; **p<0.01; ***p<0.001.

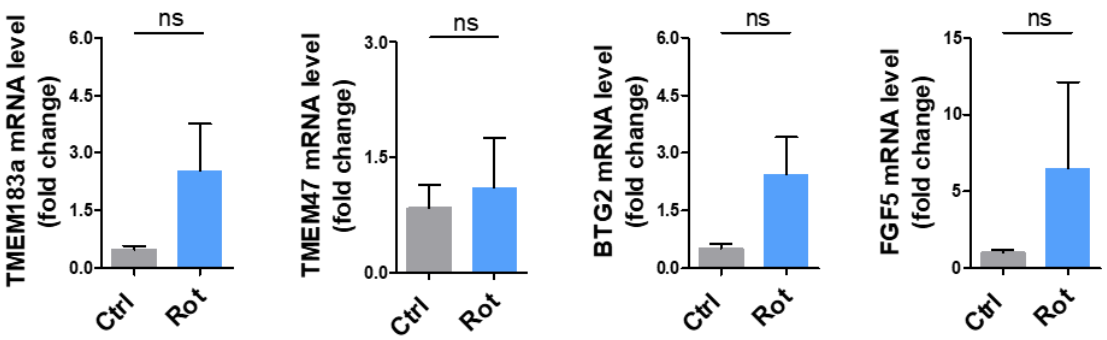

Supplementary Figure 8. Increased mRNA levels of selected genes in rotenone (Rot)-exposed midbrain slices from wild-type C57BL/6 mice. The qRT-PCR analysis of mouse midbrain slices dissected from 7- to 12-day-old pups. Four differentially expressed genes related to mitochondrial or neuronal functions were selected from the upregulated overlapping gene pool attained by integrating RNA-seq and ChIP-seq analyses. At least four independent experiments were measured. Bar graphs show mean ± SEM of unpaired two-tailed t-tests. ns, not significant; *p≤0.05.

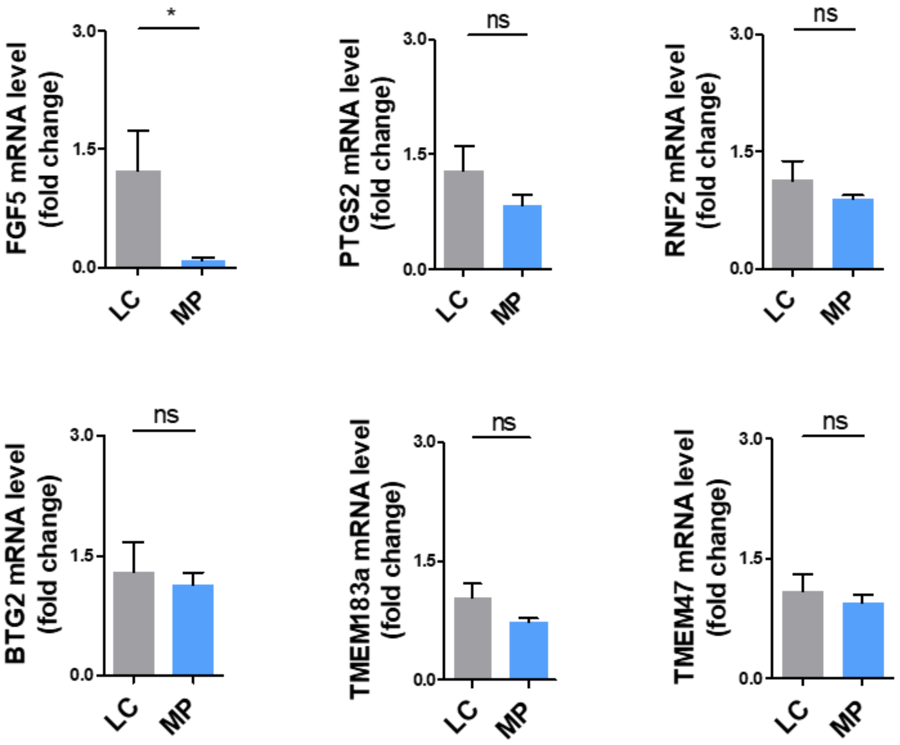

Supplementary Figure 9. Decreased mRNA levels of selected genes in the MitoPark (MP) striatum support H3K27ac accumulation in the substantia nigra, but not the striatum. Six differentially expressed genes related to mitochondrial or neuronal functions were selected from the upregulated overlapping gene pool attained by integrating RNA-seq and ChIP-seq analyses. The qRT-PCR analysis of striatum from 16- to 18-week-old littermate control (LC) and MP mice shows decreased mRNA level of selected genes. Bar graphs show mean ± SEM of unpaired two-tailed t-tests with n=4-8. ns, not significant; *$p \leq 0.05$.
